# Physical functioning limitations and physical activity of people experiencing homelessness: A scoping review

**DOI:** 10.12688/hrbopenres.13011.2

**Published:** 2021-03-01

**Authors:** Sinéad Kiernan, David Mockler, Clíona Ní Cheallaigh, Julie Broderick

**Affiliations:** 1 Discipline of Physiotherapy, Trinity College Dublin, the University of Dublin, Dublin, Ireland; 2 Department of Physiotherapy, St. James's Hospital, Dublin, Ireland; 3 John Stearne Medical Library, Trinity College Dublin, the University of Dublin, Dublin, Ireland; 4 School of Medicine, Trinity College Dublin, the University of Dublin, Dublin, Ireland; 5 Department of Clinical Medicine, School of Medicine, Trinity Translational Medicine Institute, Trinity College Dublin, Dublin, Ireland; 6 Department of Infectious Diseases, St. James's Hospital, Trinity College Dublin, Dublin, Ireland

**Keywords:** Functional status, physical activity, homeless adults, homelessness

## Abstract

**Background: **Adults who are experiencing homelessness suffer higher levels of premature mortality and age-related medical conditions compared to the general population, but little is known about physical factors that influence their health experience. The aim of this scoping review was to evaluate what is known about physical functional limitations and physical activity levels and how they are measured in adults experiencing homelessness.

**Methods: **This review was conducted in accordance with the Joanna Briggs Institute’s methodology for scoping reviews. Suitable quantitative and qualitative articles were searched using PubMed, CINAHL, EMBASE, PsychInfo, Web of Science and SCOPUS databases using a combination of keywords and a gray literature search was performed. Two reviewers independently screened articles for inclusion. Inclusion criteria were studies that examined physical functional limitations and/or physical activity among homeless adults (with/without co-occurring mental illness, infectious disease, substance use disorder), as a primary or secondary outcome measure.

**Results:** We identified 15 studies for inclusion including 2,018 participants. Studies were primarily quantitative (n=13) and there were 2 qualitative studies. The following outcomes related to physical functioning were reported; mobility levels (n=3), frailty (n=1), flexibility (n=2), strength (n=1), physical symptom burden (n=3), and exercise capacity (n=3). Eight studies reported outcomes related to physical activity. The majority of studies reported high levels of functional limitations among participants and low physical activity levels although a spectrum of abilities was noted.

**Conclusion:** This review showed that many adults who are homeless appear to show a high burden of physical functional limitations and low physical activity levels but more objective and consistent measures should be applied to examine these factors in future studies. This will help address and plan future care, physical rehabilitation and housing needs for this vulnerable cohort. This scoping review will help direct research and future systematic reviews in this emerging area.

## Introduction

The number of people experiencing homelessness is significant and increasing, with estimates of 307,000 people in the UK ^1^, 550,000 in the USA ^2^ and 235,000 in Canada ^3^ at any one point, based on data from 2017, 2016 and 2017 respectively. A ‘person experiencing homelessness’ is someone without stable housing who may live on the streets, in a shelter, in temporary accommodation, or in some other unstable or non-permanent situation ^4^.

Life expectancy is greatly reduced among people who are homeless. Recent data from the UK reports a mean age of death among people who died homeless of 45 years among men and 43 years among women, which compares with 76 and 81 years respectively, in the general population ^5^. In Ireland the median age at death for people experiencing homelessness in Dublin is devastatingly low at 44 years for males and 36 years for females ^6^. Contributing factors to lowered mortality levels are complex. People who are homeless people experience a ‘tri-morbidity’ of mental ill health, physical ill health, and addiction as well as complex interwoven factors related to social exclusion, higher rates of accidental, violent death and poor access to healthcare ^7^.

Common chronic diseases such as chronic obstructive pulmonary disease, asthma, epilepsy, heart disease and stroke are substantially more prevalent among people experiencing homelessness compared to stably housed individuals ^8^. External factors as well as chronic diseases have a multi-system effect with reported accelerated ageing ^9^ and early onset of geriatric conditions ^10^. Reflective of disease prevalence and other factors related to extreme socioeconomic deprivation, people who are homeless present for acute hospital care disproportionally compared to housed individuals ^11^.

The benefits of physical activity are well known and recent guidelines ^12^ have highlighted additional benefits of physical activity in terms of cognitive health health-related quality of life, mental health and sleep which has largely been explored in healthy populations. Information on physical activity levels among individuals who are homeless is not well known ^13^. 

Physical performance and functional limitation measures may provide an insight into early signs of disability, poor health, hospitalization and increased death risk ^9, 12^. These measures give an indication of a person’s ability to perform everyday tasks making them good indicators of overall ability to live independently as ageing occurs ^9^. To date there has been no prior effort to characterize the overall physical status of people experiencing homelessness. Improved understanding of physical functioning and physical activity is important, as this may guide the development of screening tools to identify, and interventions to attenuate declines in people experiencing homeless. This will also help direct research as well as future systematic reviews in this topic area.

The protocol was developed and peer-reviewed locally and then registered in the PROSPERO database ( CRD42019124306). In order to address the breadth of this area however, a scoping review rather than a ‘pure’ systematic review ^14^ was conducted. Although some consider a scoping review a form of systematic review ^15^, subtle differences are, for example, the breadth of the research question and the lack of risk of bias assessment ^14, 15^.

Based upon the PCC (Population, Concept and Context) elements ^16^, the overall aim of this scoping review was to evaluate the magnitude and scope of literature pertaining to the overall physical status of adults experiencing homelessness. Specific objectives were to evaluate the quantitative and qualitative literature on the following topics (i) physical functioning in adults experiencing homelessness, (ii) physical activity in adults experiencing homelessness, (iii) related secondary outcome measures such as frailty and cardiovascular fitness. In addition a further objective was (iv) to evaluate measurement methods of physical outcomes in included studies. 

## Methods

This review was informed by the Joanna Briggs Institute’s (JBI) methodology for scoping reviews ^14^ and guided by the original framework of Arksey and O’ Malley ^16^, and enhancements proposed by Levac *et al.* ^17^. This review was checked against the Preferred Reporting Items for Systematic reviews and Meta-Analyses extension for Scoping Reviews (PRISMA-ScR) Checklist ^18^ (see reporting guidelines ^19^).

### Data sources and searches

A comprehensive search strategy was developed collaboratively with a skilled research librarian (D.M.) and a subject expert (C.N.C.) was consulted. The subject expert was a medical consultant who developed an inclusion health service for adults experiencing homelessness and is the clinical lead for service provision for people experiencing homelessness admitted to a large acute inner-city hospital in Dublin, Ireland. The following electronic databases were searched without date restrictions; MEDLINE/PubMed, EMBASE, PEDro, AMED, CINAHL, PsycINFO, SCOPUS (see extended data ^19^). A grey literature search using Google Scholar and WorldCat search engines was performed; government reports were searched using the Google search engine and a combination of key word text from inception to 16.01.19.

### Physical focused definitions employed in this review

We employed Nagi’s ^20^ definition of functional limitations as restrictions in the basic performance of the person such as limitations in the performance of locomotor tasks, such as the person’s gait, and basic mobility. Although not the specific focus of this review, factors that relate to physical functioning limitations such as, but not limited to, frailty, physical symptom burden and cardiovascular fitness were included if reported in studies sourced. Physical activity was defined as any bodily movement produced by skeletal muscles that results in energy expenditure ^21^ and was considered any type of physical training or movement, including any form of exercise, physical fitness or therapeutic movement. The full search strategy is available in Supplementary File 2.

### Inclusion/exclusion criteria

This review included English language studies only. To meet the objective of the scoping review questions in this study, both qualitative and quantitative study designs were included. Studies that examined physical functioning or physical activity (separate searches for each were conducted and later combined) among homeless adults (>18 years) as a primary or secondary outcome measure were included. The following criteria for homeless from the European Typology for Homelessness and Housing Exclusion (ETHOS) criteria ^22^: roofless, houseless, living in insecure housing, living in inadequate housing was employed in this review.

### Selection of studies

Duplications were removed and relevant studies were imported into Covidence for title and abstract screening which took place independently by two reviewers (J.B. and S.K.). Both authors then conducted a full-text evaluation of selected studies. If necessary, any discrepancies were resolved by consensus by including a third author (C.N.C.).

### Data extraction

Two reviewers (S.K. and J.B.) independently extracted data using a specifically designed data extraction sheet. The data extraction instrument collected the following data relating to included studies (author, year of publication, country of study origin, inclusion criteria, living arrangements, physical focused outcomes measured, participant characteristics (number of participants, age, biological sex, race/ethnicity, percentage with less than high school education, co-morbid conditions), physical focused variables (physical variable measured, type of measure, total number of studies, authors, results), physical activity/sedentary behavior focused measures (author, type of measure, measure subscale, main results). Any differences were resolved by consensus discussion. A third author (C.N.C) was available if disparities emerged between reviewers.

### Data analysis

Descriptive analysis was performed for all demographic data and data was grouped according to outcome evaluated. Due to the heterogeneity of study design, interventions and outcomes, a narrative synthesis was conducted.

## Results

### Studies identified

**Figure 1.**
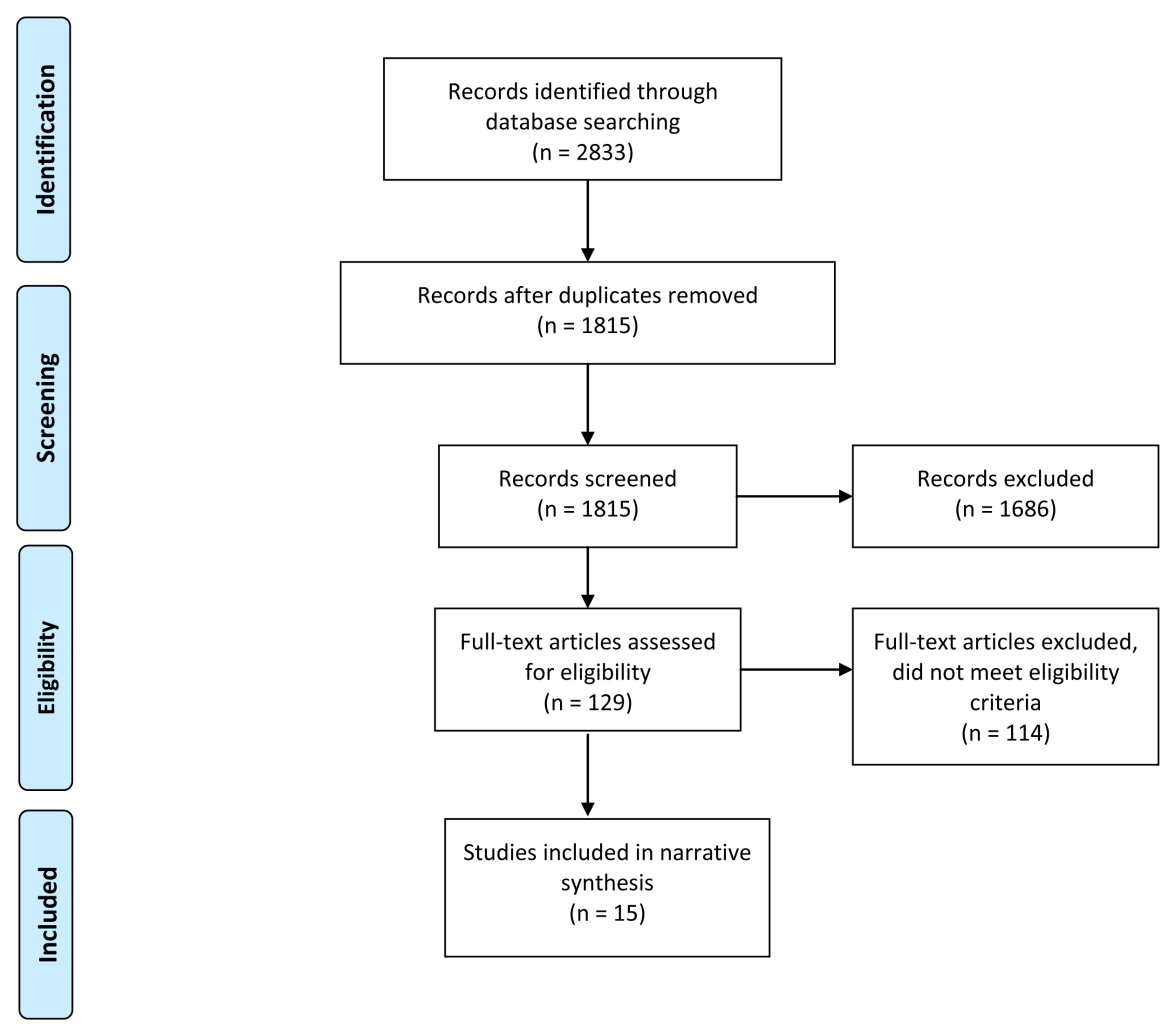
PRISMA flow diagram of selection for review.

After the removal of duplicates, 2832 studies were identified. After excluding studies which did not containing data relevant to physical functioning limitations or physical activity specific to adults who were homeless, a total of 15 studies were deemed eligible for inclusion in this reviewAfter excluding studies which did not containing data relevant to physical functioning limitations or physical activity specific to adults who were homeless, a total of 15 studies were deemed eligible for inclusion in this review. The PRISMA flow chart ^23^ summarizes the search strategy ( Figure 1). Quantitative (n=13) studies predominated and the remaining were qualitative in design (n=2). Over 2000 participants were included in this review (n=2,018). Over 70% of participants were male. A formal operational definition of homeless was included in one study only ^24^. The living arrangement of participants was outlined in the recruitment strategy and/or eligibility criteria of remaining studies. The majority of studies included participants in shelter accommodation. Four studies were limited to male only participants ^24– 27^, while only two were female only ^28, 29^. Characteristics of the included studies are shown in Table 1. The majority of studies took place in North America (12/15) with the remainder in Australia (n=1) and Denmark (n=2).

**Table 1.** Study characteristics.

Author and year	Study Location	Listed study type	Inclusion criteria	Definition of homelessness/living arrangement/access to services	Physical- functioning measure or Physical activity measure	Outcomes (measure)
**Quantitative Studies**						
Ballard, 2009	North Carolina, U.S.	Cross sectional	Age >18 years Understood and spoke English	Staying overnight in a shelter got homeless women	Physical activity	-Physical activity subscale of the Health Promoting Lifestyle Profile II questionnaire
Brown *et al.* 2012	Boston, U.S.	Cross sectional	Age >50 years Able to communicate in English Able to give consent	Accessing emergency, transitional and day centers	Physical functioning	-Frailty (Fried frailty criteria, -Functional status (self-reported mobility impairments)
Brown *et al.* 2017	California, U.S.	Prospective cohort study	Age >50 years Able to give consent English speaking Homeless	Accessing low-cost meal programs serving (for homeless people), overnight shelters, also ‘recycling centers and places where unsheltered people stayed’	Physical functioning	-Physical activity (self- reported frequency)
					Physical activity	-Functional status (self-reported mobility impairments)
Chau *et al.* 2002	Los Angeles, U.S.	Survey	Homeless English-speaking >18 years New to study	No regular home or apartment to stay in for at least one night of the previous 30 nights but had to stay in: ( *a*) a shelter; ( *b*) a hotel paid for with a voucher; ( *c*) the street or other outdoor public place; ( *d*) a church; ( *e*) an indoor public place; ( *f*) an abandoned building; or ( *g*) a car or other vehicle	Physical activity	-Daily exercise habit (self- report)
Gaderman *et al.* 2014	Vancouver, Toronto, Ottowa, Canada	Cross sectional	Age >18 years	Accessing homeless shelters, meal programs, rooming houses, and a supervised injection site	Physical functioning	-Physical health impact (Short form-12 questionnaire)
Gregg and Bedard 2016	Winnipeg, Canada	Cross sectional	Not specified	Patrons of a homeless shelter	Physical activity	Reporting of frequency of exercise
					Physica functioning	-Fitness (1 mile treadmill walk test) -Strength (grip strength) -Flexibility (sit and reach test)
Kendzor *et al.* 2015	Dallas, U.S.	Pilot study	>6th grade English literacy, Willingness to quit smoking Age >18 years Willingness to attend weekly smoking cessation treatment sessions	Patrons of a homeless shelter	Physical activity	-Physical activity (7 items from the Behavioral Risk Factor Surveillance System Questionnaire)
Marmolejo *et al.* 2018	Los Angeles, U.S.	2 group cross-sectional comparative study	Ability to give consent	Accessing a homeless youth drop-in centre	Physical activity	-Self-report physical activity questionnaire)
					Physical functioning	-Flexibility (sit and reach test)
Patanwala *et al.* 2017	California, U.S.	Cross-sectional analysis within longitudinal cohort study	Age > 50 years English-speaking Defined in the federal Homeless Emergency Assistance and Rapid Transition to Housing Act Able to give informed consent	Accessing low-cost meal programs serving (for homeless people), overnight shelters, also ‘recycling centers and places where unsheltered homeless adults stayed’	Physical functioning	-Physical symptoms (Patient Health Questionnaire 15)
Randers *et al.* 2010	Copenhagen, Denmark	Cross sectional	NS	Recruited from shelters and unemployment offices	Physical functioning	-Fitness (VO _2_max test)
Randers *et al.* 2012	Copenhagen, Denmark	Controlled study	NS	Recruited from shelters and unemployment offices	Physical functioning	-Fitness (VO _2_max test)
Raven *et al.* 2017	California, U.S.	Cross sectional	English speaking Age > 50 years	Recruited from people in homeless encampments, one recycling centre, all overnight homeless shelters and free and low cost meal programmes	Physical functioning	-Functional limitations (Short physical performance battery)
Wilson, 2004	Midwest, U.S.	Cross-sectional study	Homeless women Registered residents of the shelters Could read and understand the English language	Recruited from urban shelters	Physical activity	-Physical activity levels (Health-Promoting Lifestyle Profile II questionnaire)
**Qualitative studies**						
Bazari *et al.* 2018	California, U.S.	Qualitative study including semi-structured interviews	Age >50 years Able to give consent English speaking Homeless	Recruited from low-cost meal programs, recycling centers, overnight homeless shelters and locations where unsheltered adults stay	Physical functioning	-Physical symptom burden (semi-structured interviews)
Quine *et al.* 2004	Sydney, Australia	Qualitative study	Older men ≥ 50 years, In receipt of a pension or benefit Effectively single Non-home owners Living alone	Non home-owning men living alone in insecure housing in inner city area	Physical activity	-Physical activity levels (semi structured interviews)

Participant characteristics are shown in Table 2. Despite the relatively low mean/median age of participants [2 ^nd^ decade (n=2 studies), 3 ^rd^ decade (n=2 studies), 4 ^th^ decade (n=5 studies), 50 ^th^ decade (n=5 studies), 60th decade, (n=2 studies), participants experienced a high burden of physical and mental conditions. From data presented in included studies, rates of hypertension ranged from 20.4% to 59%, arthritis from 16.8% to 46.8%, diabetes from 14% to 18.3% and depression from 34% to 59.6%.

**Table 2.** Details of participant characteristics.

Citation	Number of participants	Age mean (SD)	Biological Sex	Race/ Ethnicity	<High school education	Comorbid conditions
Ballard, 2009	126	41.99 ± 9.42 years	Female only M:0 F:126	African American (54%) White (32.5%) American Indian (4.8%) Mixed race (4.8%) Asian (1.6%) Other/unsure (4.4%)	31.8%	High blood pressure: 41.1% Asthma: 26.8% Arthritis: 25% STDs: 22.4%
Bazari *et al.* 2018	20	62 years	Male= 65% M:13 F:7	African American (85%)	NS	NS
Brown *et al.* 2012	247	56 years	Male= 92% M:187 F:60	White (39.7%)	26.1%	Hypertension (59%), arthritis (44.9%), depression (59.6%)
Brown *et al.* 2017	350	58 (54–61 years) ^a^	Male= 77.1%	African American (79.7%), White (10.9%) Latino (4.6%), Other (4.9%)	25.7%	Hypertension (56%) Coronary artery disease or myocardial infarction (9.1%) Congestive heart failure (7.1%) Diabetes (14%) Stroke (11.2%) Respiratory disease (26.3%) Arthritis (44.6%) HIV/AIDS (5.5%)
Chau *et al.* 2002	221	46.7 years	Male=54% M:120 F:101	African-American (57%) Caucasian (26%) Other (17%)	60%	NS
Gadermann *et al.* 2014	100	43.3 +/- 11.9 years	Male= 69% M:69 F:31	White (55%), Aboriginal (30%) Other (15%)	27.2%	Arthritis/rheumatism, joint problems (43.9%), Hepatitis C (31.6%), Migraines (28.6%), Mental health conditions (52.5%), Substance abuse (40.2%), Depression (34%), Substance dependence (26.6%), GAD (15.6%), PTSD (12.5%)
Gregg and Bedard 2016	18	41.05 ± 11.32 years	Male = 100% M:18 F:0	NS	NS	NS
Kendzor *et al.* 2015	57	49.4 +/- 7.7 years	Male = 66.6%	African-American (54.4%) Latino (3.5%) Mixed race(5.3%)	NS	NS
Marmolejo *et al.* 2018	40	21.4 ± 2.3 years	Male = 67.5% M:27 F:13	White (30%) Hispanic (27.5%) African American (20%) American Indian/ Alaska Native 3(7.5%) Native Hawaiian /Pacific Islander 1(2.5%) Missing (12.5%)	15%	NS
Pantalawa *et al.* 2017	283	59 (51–82) ^a^	Male=75.6% M:214 F:69	African American (82.4%) White (9.6%) Other (21.9%)	21.9%	Heart related (17.2%) Respiratory related (23.7%) Diabetes (18.3%) Arthritis (46.8%) Cirrhosis/liver disease (21.0%) Kidney disease (5.4%) Cancer (5.9%) HIV/AIDS (6.2%)
Quine *et al.* 2004	32	66 years	Male = 100% M:32, F:0	Australian born (66%) Born overseas (33%)	NS	‘Significant’ health difficulties (66%)
Randers *et al.* 2010	15	29 ± 2 years	Male = 100% M:15,F:0	NS	NS	NS
Randers *et al.* 2012	22	37 ± 10 years	Male = 100% M:22, F:0	NS	NS	NS
Raven *et al.* 2017	350	58 (54–61) ^a^	Male = 77.1% M:270 F:80	African American (79.7%) Non-African American (20.3%)	74.3%	Chronic illness (23.9%), Acute illness (21.6%), Pain (19.2%) PTSD (32.6%) Depression (53.3%)
Wilson, 2004	137	36 years (range 18–60)	Female only M:0 F:137	White (53%) African American (43.8%)	22%	Physical diseases: Asthma: 27% Chronic bronchitis: 25.5% Hypertension: 20.4% Arthritis: 16.8% STD: 16.8% Ulcer: 15.3%

NS: not stated, ^a^Median(IQR), Abbreviations: AIDS; acquired immunodeficiency syndrome, GAD; generalised anxiety disorder, HIV; human immunodeficiency virus, F: female, M; male, NS; not stated, PTSB; post-traumatic stress disorder, STD; sexually transmitted disease,

The following physical variables were evaluated in studies included in this review; mobility status, frailty, flexibility, physical symptom burden, physical activity levels and exercise intensity achieved and fitness. Table 3 summarizes physical focused variables and Table 4 summarizes physical activity/sedentary behavior variables.

**Table 3.** Physical focused variables measured in systematic review studies.

Physical Variable	Type of Measure	Total number of studies	Authors
Mobility	Self-reported difficulty walking	2	Brown *et al.* (2012) Brown *et al.* (2016)
Lower extremity functioning	Short Physical Performance Battery	3	Raven *et al.* (2017)
Frailty	Fried criteria	1	Brown *et al.* (2012)
Flexibility	Sit and Reach Test	1	Marmolejo *et al.* 2018 Gregg and Bedard (2016)
Strength	Grip Strength	1	Greg and Bedard (2016)
Physical health/ symptom burden	Physical symptom burden (self-report)	1	Bazari *et al.* (2018)
	SF-12 (Physical component)	1	Gaderman *et al.* (2014)
	Patient Health Questionnaire-15	1	Pantanwala *et al.* (2017)
Exercise capacity	1 mile walk test	1	Greg and Bedard (2016)
	V0 _2_max	2	Randers *et al.* (2010) Randers *et al.* (2012)

**Table 4.** Physical activity/sedentary behaviour focussed measures.

Author	Type of measure	Detail of measure	Subscale (if relevant)	Main Result
Ballard, 2009	Questionnaire	Health Promotion Model Measures	Physical activity subscale [Health-promoting Lifestyle Profile II (HPLP II)]	2.08 (0.66) Range: 1.00–3.88
Chau *et al.* 2002	Interview	Asked in interview if exercise was ‘daily’, ‘sometimes’ or ‘never’	N/A	125 (56%) exercised daily, 86 (39%) exercised sometimes, 10 (5%) never exercised
Gregg & Bedard, 2016	Reporting of frequency of exercise	Exercise defined as ‘’at least three times per week, for at least 20–30 min in duration, and at least moderate-to- vigorous intensity’’	N/A	8 (44%) participants reported exercising regularly
Kendzor *et al.*, 2015	Questionnaire	Behavioural Risk factor Surveillance System Questionnaire	Insufficient physical activity defined as <150 minutes of moderate physical activity or <75 minutes of vigorous physical activity (or less than an equivalent combination of the two)	During the previous week, 26.3% did not meet recommended physical activity guidelines
Marmolejo *et al.* 2018		Self-report paper questionnaire but unclear exactly how physical activity measured	‘Low frequency’ physical activity 0–2 times per week	N=14, 36.8%
			‘High frequency’ Physical activity 3+ times/week	N=24, 63.2%
Quine *et al.* (2004)	Self-report	Semi-structured interview	N/A	Physical activity (walking) emerged as a theme
Wilson	Questionnaire	Health Promotion Model Measures	Physical activity subscale [Health-promoting Lifestyle Profile II (HPLP II)]	2.05 (+/-0.98)

N/A: not applicable

### Mobility status

Mobility status was evaluated in two studies. Overall results indicated that many people homeless experiencing homelessness have difficulty mobilizing. In two studies ^10, 30^ mobility was measured by self-reported difficulty walking. Brown *et al.* 2012 ^30^ sampled 247 homeless adults, and found that 102 (41.3%) self-reported difficulty walking ^30^. Brown *et al.* 2017 included 350 participants aged 50 or older and reported mobility impairments in over one quarter of participants (26.9%) and 33.7% reported one or more falls in the previous 6 months. Results of this study indicated that greater mobility impairments (defined as difficulty across a room) were found in participants < 50 years, compared to those ≥ 50 years.

### Functional limitations

Raven *et al.* 2017 reported that over half (58.4%, n=204) of participants had limitations in lower extremity function measured by the Short Physical Performance Battery ^31^. This study included participants with a median (IRQ) age of 58 (54–61) years.

### Frailty

Frailty was evaluated in one study ^30^. Frailty was measured using the Fried criteria ^32^ in which more than 3 of 5 characteristics were present: unintentional weight loss, low physical activity, exhaustion, slow walking speed and weak handgrip. In total, 40 participants (16%) met frailty criteria, bearing in mind that participants were aged between 50 and 69.

### Flexibility

Flexibility was assessed in two studies ^24, 33^ and compared to control groups. The Sit and Reach test ^34^ was used which targets hamstring and lower back flexion. Other flexibility tests employed were the butterfly test (targets adductor muscles), the trunk flexibility test and shoulder stretch ^34^. Mean (SD) results for the sit and reach test, butterfly test, left shoulder, right shoulder, left trunk twist and right trunk twist were 26.2 (9.01), 17.83 (7.29), 0.59 (9.55), 2.42 (7.54), 8.89 (7.96), 12.22 (8.23) respectively ^33^. It was noted that participants who were homeless were less flexible (p<0.05) in four stretch tests compared to a control group of university students. Similar low values were reported for the Sit and Reach test in the Gregg and Bedard (2016) ^24^ study of 24.32 ± 8.07cm.

### Strength

Strength was measured in one study ^24^ using a grip strength test ^35^ which was reported to be mean (SD) 43.24 (6.79). Values from the homeless cohort age 41.05 ± 11.32 years were reported to be comparable to a reference population.

### Physical health/symptom burden

Physical symptom burden was evaluated in three studies, assessed in 3 different ways. Patanwala *et al.* (2017) evaluated physical symptoms in homeless aged ≥ 50 years ^36^ using the Patient Health Questionnaire-15 (PHQ-15) ^37^. They reported that over one-third (34%, n= 96) had a moderate-high physical symptom burden. The most common physical symptoms were joint pain, fatigue, back pain and sleep difficulties.

Similarly, Gaderman *et al.* (2014) using the SF-12 ^38^, reported that the physical component summary scale was 43.6 (SD=11.0), which was ‘substantially lower’ than US population normative values ^39^. In this study is was found that 87.9% (n=53) of participants suffered at least one physical health condition.

These findings concur with a qualitative study included in this review. Bazari *et al.* (2018) reported that physical symptoms experienced by homeless adults interfere with daily functioning ^40^. They included 20 participants aged between 52 and 78 years (median age 62). It was found that daily challenges and physical conditions of homelessness caused and exacerbated symptoms.

*“I can’t be active anymore like playing sports because I used to like to go play basketball or lift weights… but I can’t do nothing anymore…”* (M, 63) **

Some participants cited premature aging as the reason for their physical symptoms and decreased functional ability.

*“It’s the arthritis…. Sometimes I feel I am carrying all my weight on my legs….I just feel like I’ve aged so quickly in my life”* (F, 58)

Fatigue was also a factor.

*‘’I guess every day that I have to walk I’m tired. I guess that’s the main thing: that I go from bench to bench and feel tired’’* (M, 58)

### Physical activity levels

Physical activity levels were measured in six studies. Diverse methods were employed to assess this construct in each study. Insufficient physical activity levels among homeless adults were generally reported across studies ( Table 4). Kendzor *et al.* (2015) examined modifiable health risk factors among homeless smokers (n= 57) ^41^. The results showed that 26.3% did not meet recommended physical activity levels in the previous week. Chau *et al.* 2002 asked about exercise habits during an interview which mainly focused on cancer risk behaviours and screening. It was reported that 56% (n=125) performed daily exercise, but no details of the definition of exercise was supplied. Gregg and Bedard (2016) evaluated ‘regular exercise’ as per Courneya and Bobick, 2000 ^42^ and reported that 44% (n=8) exercised ‘’at least three times per week, for at least 20–30 min in duration, and at least moderate-to-vigorous intensity’’. Wilson (2005) explored health-promoting behaviours of women who were living in shelter accommodation (n= 137) ^29^. The study employed the Health-Promoting Lifestyle Profile II (HPLPII) ^43^ and found that participants scored lowest in the physical activity subscale which is shown in Table 5 although overall it was reported that total levels of health-promoting behaviours were similar to another study of low income and homeless women ^44^.

**Table 5.** Health-Promoting Lifestyle Profile - Physical activity subscale.

Health-Promoting Lifestyle Profile - Physical activity subscale (From Wilson, 2004)	Mean (SD)
Follow a planned exercise program	1.78 (0.77)
Exercise vigorously for 20 or more minutes at least three times a week (such as brisk walking, bicycling, aerobic dancing, using a stair climber)	2.05 (0.98)
Take part in light to moderate physical activity (such as sustained walking 30–40 minutes 5 or more times a week)	2.28 (0.93)
Rake part in leisure-time (recreational) physical activities (such as swimming, dancing, bicycling)	2.02 (0.76)
Do stretching exercises at least 3 times per week	1.90 (0.89)
Get exercise during usual daily activities (such as walking during lunch, using stairs instead of elevators, parking away from destination and walking)	2.59 (0.94)
Check my pulse when exercising	1.53 (0.80)
Teach my target heart rate when exercising	1.61 (0.76)

Quine *et al.* (2004) ^27^ employed semi structured interviews and a number of facets of physical activity emerged. It found that some participants were until recently physically active. However, deterioration in their health had reduced their activity levels.

*“I used to walk about a quarter of a mile up and around the block”* (M, 86)

Physical activity was also undertaken as a necessity.

*‘’It’s a good walk [to a meals centre] and they put on a hot breakfast’’* (M, 68)

Physical activity was also used as a time filler

*‘’if there’s something on like a movie worthwhile I’ll watch that and if there’s not I’ll for out for a walk for an hour and come back’’* (M, 75).

### Exercise capacity

Randers *et al.* (2010) reported VO _2_ max levels for 15 people experiencing homelessness who were engaging in a football training program. Reported VO _2_ max levels were 33.5 +/- 2.0 ml.kg.min ^-1^ ^25^. Similarly, Randers *et al.* 2012 reported VO _2_ max levels for 22 men experiencing homelessness before and after a 12 week soccer training program. Reported VO _2_ max levels were 36.7 +/- 7.6 ml.kg.min ^-1^ which appeared higher than a control group (33.7 +/- 4.5) ^45^. One further study evaluated fitness using the 1 mile walk test ^24^ with a result of 16.48 +/- 2.42 minutes which was reported to be similar to reference values for age and gender.

## Discussion

This review provided a snapshot of existing literature in the area of physical functioning limitations and physical activity levels in people experiencing homelessness. The scoping review methodology enabled a broad range of inter-related physical related variables (mobility status, functional levels, frailty, flexibility, physical symptom burden, physical activity levels and exercise capacity) to be usefully subsumed into one review which gives a broad overview of this topic area. It is clear from this review that the experience of homelessness negatively influences physical –focused parameters but the diversity of measures limited our ability to synthesize data for the purposes of this review.

This review included 2,018 participants, of which females were underrepresented as over 70% of review participants were male. This reflects that 4 studies exclusively included males, whereas only 2 studies only included females, and relatively there was a higher proportion of males than females in the remaining studies. Less therefore appears to be known about the physical profile of females experiencing homelessness compared to males. Sex as a biological characteristic was reported in studies was reported rather than gender which is more a social and identity construct ^46^. It is known that transgender people are disproportionally represented among homeless populations ^47^ but this group were not represented in studies included in this review. 

The majority of studies included in this review were quantitative in design (n=11), while 3 were qualitative. Almost 80% of studies were based in North America, with the rest of studies from other high income countries of Denmark and Australia. There appears to be a large evidence gap in the evaluation of physical variables among people in low and middle income countries.

In the US based studies 59.6% of participants were African American, while a lower proportion were white (29.8%). This reflects the high proportion of African Americans among homeless populations in the US ^48^. Indigenous people are also over-represented among homeless populations ^49^ which likely mirrors the proportion of Aboriginal people in a Canadian study ^42^ included in this review. It is possible that in other studies this group may have been under-represented or not specifically reported. Out of 10 US based studies, one reported the proportion of American Indian participants was 4.8%, and another quoted that 10% of participants were American Indian/Alaska Natives/Native Hawaiian/Pacific Islanders. Most of the rest of the studies included categories of ‘other’ in which it was likely native populations were subsumed. Similarly, there may have been an under-representation of Latino people and people of mixed race heritage but absolute proportions of different ethnic groups among homeless populations are likely to be context specific. 

Studies predominately appeared to include people in shelter accommodation. The proportion of people sleeping rough who were included in studies within this review was low and it is probable that their physical health variables may be worse than individuals living in sheltered accommodation. Despite the frequency of hospital visits and stays in this population ^11, 50^, no study profiled hospitalized homeless individuals. It is likely that this cohort may be especially vulnerable and debilitated and requires further evaluation with regard to physical focused variables.

Despite the disparity in measures, there generally appears to be a pattern of low physical functioning levels and poor physical activity levels among people experiencing homelessness compared to expected levels. A high physical symptom burden was also noted particularly in relation to joint pain, fatigue, back pain and sleep problems ^36^. Flexibility levels were also significantly lower than control group findings ^33^. This finding suggests a global decline or substandard level of physical fitness and function among homeless adults and an earlier onset of geriatric conditions which has been shown previously ^51^, the reasons for which need to be further elucidated. In the study by Brown *et al.*, 2017, it was noted that despite a median age of 58 years, participants had rates of geriatric conditions similar or equivalent to adults in the general population with a median age of nearly 80 years ^52, 53^. Similarly, the study by Raven *et al.* included participants with a median age of 58 years and reported that almost 60% had limitations in lower extremity function. This was also shown in the earlier study by Brown ^30^ and provides more evidence for the need for geriatric style rehabilitation services needed for people experiencing homelessness ^10^.

At odds with the majority of studies, two Danish studies ^25, 26^ which evaluated fitness in a population of people experiencing homelessness who were participating in street soccer showed comparable fitness levels to control group values but mean ages were in the 3 ^rd^ decade in these studies. Gregg and Bedard also showed that fitness and strength were comparable to reference ranges among healthy populations ^54^ in also a relatively young cohort with an average age of 41.05 +/- 11.32 years. It is possible that these groups are not representative of the population as a whole, nonetheless the diversity of people experiencing homelessness and spectrum of ability is important to consider. It is also possible that physical functioning limitations may develop after the 3 ^rd^ and 4 ^th^ decades for some people experiencing homelessness.

While reported physical activity levels varied between studies, a large proportion of participants experiencing homelessness appeared to have low physical activity levels ^33^. Promoting physical activity may mitigate against some of the burden of physical and mental health issues suffered by people experiencing homelessness ^46^. One study ^27^ highlighted a nuanced view indicating that physical activity was undertaken not necessarily for health gain but by participants out of necessity to access meals and to fill in time.

The number of outcomes and measures suggests a lack of empirical data in the area to aid clinical decision makers and researchers about the overall physical health status of people experiencing homelessness. Physical focused measures included in this review were for the most part cursory in nature and were subsidiary to other study outcomes. While a diversity of outcomes were included in studies included in this review, self-report measures were predominantly used rather than more robust objective methods with the exception of two studies which employed a gold standard measure to evaluate V0 _2_ max ^32, 33^. Studies by Brown *et al.* (2011), Brown *et al.* (2017) and Raven *et al.* (2017) were the only studies to examine mobility impairment. Only one study used the Short Physical Performance Battery, a useful battery of physical performance tests to assess functional status ^47^. Only one study evaluated frailty and falls (Brown *et al.* 2011). All studies which evaluated physical activity used self-report measures which lack reliability and are prone to inaccuracies ^48^.

The general lack of robust data which extensively evaluates physical functioning and physical activity among people experiencing homelessness may be also partly due to concerns regarding vulnerability and potential or perceived ability to participate in research can result in exclusion from research. This can lead to a lack of evidence on which to base policies and design suitable housing services.

## Strengths and limitations

This review appears to be the first attempt to systematically present literature pertaining to physical functioning limitations and physical activity levels in adults experiencing homelessness. The scoping review methodology employed in this review was suitably broad to bring together evidence from heterogeneous methodology sources including observational, mixed method and qualitative designs of the experience of physical limitations in people experiencing homelessness as well as the diverse reporting of outcomes ^55^. This scoping review allowed various inter-related physical aspects such as frailty, cardiovascular fitness, and flexibility among others. This methodology was also useful to examine emerging evidence in this relatively new field of research. In a topic as broad as physical functioning limitations it has helped focus on where future research and eventual systematic reviews should be targeted.

A number of limitations pertained to this review, however. Firstly, studies lacked a consistent definition of homelessness. As diverse study designs were included in this review, this resulted in strong heterogeneity which precluded the ability to quantitatively analyse results. A formal assessment of methodological quality of the included studies was not performed as scoping reviews aim to include a broad overview of available evidence, irrespective of quality ^55^. Finally, potentially relevant evidence from other languages may have been missed as this review only included English language papers.

As all studies included in this review were community based, the generally low level of physical functioning and physical activity of this population is relevant to a broad spectrum of community based services including housing, social health services. Housing services should bear accessibility in mind and social activities should incorporate a physical/exercise component where possible. 

Bearing in mind the prevalence of physical functioning limitations, we would advocate that all clinicians should screen this population for physical deficits so appropriate rehabilitation or other services can be initiated. We appreciate however, that the non-uniformity of outcomes and measurement tools applied presents a challenge to clinicians. Recommendations on appropriate physical functioning and physical activity measures are needed which are suitable to use in this population to prevent waste of valuable healthcare resources ^49^. Studies should focus on reliability, validity and responsiveness of physical functioning measures for people experiencing homelessness as a basis for more effective clinical assessment and management. Further research should determine a core outcomes set ^56^ applicable to this population. Ideally this would be a quick standardized physical test battery so reliable consistent data can be collated to highlight at risk groups, inform clinical decision making and practice and advocate for better services. Further consistent primary research needs to be conducted before a comprehensive systematic review can be conducted. Factors possibly contributing to physical functioning limitations such as age, co-morbidities as well as a host of other factors also need further exploration.

## Conclusion

This review shows that adults experiencing homelessness appear to suffer physical functioning limitations and low physical activity levels but the inconsistency in measurement methods limits our ability to extensively profile this population at this time. Given the low levels of physical functioning shown in people experiencing homelessness, greater prominence and robustness of measurement methods should be applied to fully interrogate this area. Further research is necessary so adequate rehabilitation regimes and support can be put in place for this vulnerable population. This scoping review will guide future research and systematic review development in this emerging area.

## Data availability

### Underlying data

All data underlying the results are available as part of the article and no additional source data are required.

### Extended data

Open Science Framework: Physical functioning limitations and physical activity of people experiencing homelessness: A review. https://doi.org/10.17605/OSF.IO/7VGZP ^19^

This project contains the following extended data:

- Supplementary File 2 Search Strategy - Copy.docx (Study search strategy)

### Reporting guidelines

Open Science Framework: PRISMA-ScR checklist for ‘Physical functioning limitations and physical activity of people experiencing homelessness: A scoping review’. https://doi.org/10.17605/OSF.IO/7VGZP ^19^

Data are available under the terms of the Creative Commons Zero "No rights reserved" data waiver (CC0 1.0 Public domain dedication).

## References

[1] Shelter: Far from alone: homelessness in Britain in 2017.

[2] National Alliance to End Homelessness: State of homelessness.2018. Reference Source

[3] Gaetz S Dej E Richter T: The state of homelessness in Canada 2016. Reference Source

[4] National Health Care for the Homeless Council: Official definition of homelessness. Reference Source

[5] Office for National Statistics: Deaths of homeless people in England and Wales: 2018.

[6] Ivers JH Zgaga L O'Donoghue-Hynes B: Five-year standardised mortality ratios in a cohort of homeless people in Dublin. *BMJ Open.* 2019;9(1):e023010. 10.1136/bmjopen-2018-023010 30782692 PMC6352814

[7] Fitzpatrick S Bramley G Johnsen S: Pathways into multiple exclusion homelessness in seven UK cities. *Urban Stud.* 2012;50(1):148–68. 10.1177/0042098012452329

[8] Lewer D Aldridge RW Menezes D: Health-related quality of life and prevalence of six chronic diseases in homeless and housed people: a cross-sectional study in London and Birmingham, England. *BMJ Open.* 2019;9(4):e025192. 10.1136/bmjopen-2018-025192 31023754 PMC6501971

[9] Fazel S Geddes JR Kushel M: The health of homeless people in high-income countries: descriptive epidemiology, health consequences, and clinical and policy recommendations. *Lancet.* 2014;384(9953):1529–40. 10.1016/S0140-6736(14)61132-6 25390578 PMC4520328

[10] Brown RT Hemati K Riley ED: Geriatric Conditions in a Population-Based Sample of Older Homeless Adults. *Gerontologist.* 2017;57(4):757–66. 10.1093/geront/gnw011 26920935 PMC5881727

[11] Ni Cheallaigh C Cullivan S Sears J: Usage of unscheduled hospital care by homeless individuals in Dublin, Ireland: a cross-sectional study. *BMJ Open.* 2017;7(11):e016420. 10.1136/bmjopen-2017-016420 29196477 PMC5719262

[12] Penninx BW Ferrucci L Leveille SG: Lower extremity performance in nondisabled older persons as a predictor of subsequent hospitalization. *J Gerontol A Biol Sci Med Sci.* 2000;55(11):M691–7. 10.1093/gerona/55.11.m691 11078100

[13] Taylor A Murillo R Businelle MS: Physical activity and sleep problems in homeless adults. *PLoS One.* 2019;14(7):e0218870. 10.1371/journal.pone.0218870 31276513 PMC6611579

[14] Munn Z Peters MDJ Stern C: Systematic review or scoping review? Guidance for authors when choosing between a systematic or scoping review approach. *BMC Med Res Methodol.* 2018;18(1):143. 10.1186/s12874-018-0611-x 30453902 PMC6245623

[15] Lockwood C Dos Santos KB Pap R: Practical Guidance for Knowledge Synthesis: Scoping Review Methods. *Asian Nurs Res (Korean Soc Nurs Sci).* 2019;13(5):287–94. 10.1016/j.anr.2019.11.002 31756513

[16] Arksey H O'Malley L: Scoping studies: towards a methodological framework. *Int J Soc Res Methodol.* 2005;8(1):19–32. 10.1080/1364557032000119616

[17] Levac D Colquhoun H O'Brien KK: Scoping studies: advancing the methodology. *Implement Sci.* 2010;5:69. 10.1186/1748-5908-5-69 20854677 PMC2954944

[18] Tricco AC Lillie E Zarin W: PRISMA Extension for Scoping Reviews (PRISMA-ScR): Checklist and Explanation. *Ann Intern Med.* 2018;169(7):467–73. 10.7326/M18-0850 30178033

[19] Broderick J Kiernan S Cheallaigh CN: Physical functioning limitations and physical activity of people experiencing homelessness: A review.2020. 10.17605/OSF.IO/7VGZP PMC7934094 33728397

[20] Nagi SZ: An epidemiology of disability among adults in the United States. *Milbank Mem Fund Q Health Soc.* 1976;54(4):439–67. 10.2307/3349677 137366

[21] Caspersen CJ Powell KE Christenson GM: Physical activity, exercise, and physical fitness: definitions and distinctions for health-related research. *Public Health Rep* 1985;100(2):126–31. 3920711 PMC1424733

[22] Edgar B Meert H: Fourth review of statistics on homelessness in Europe: The ETHOS Definition of Homelessness. Belgium, Homeless; EFoNOWwt.2005. Reference Source

[23] Moher D Liberati A Tetzlaff J: Preferred reporting items for systematic reviews and meta-analyses: the PRISMA statement. *J Clin Epidemiol.* 2009;62(10):1006–12. 10.1016/j.jclinepi.2009.06.005 19631508

[24] Gregg MJ Bedard A: Mission Impossible? Physical Activity Programming for Individuals Experiencing Homelessness. *Res Q Exerc Sport.* 2016;87(4):376–81. 10.1080/02701367.2016.1233314 27736368

[25] Randers MB Nybo L Petersen J: Activity profile and physiological response to football training for untrained males and females, elderly and youngsters: influence of the number of players. *Scand J Med Sci Sports.* 2010;20 Suppl 1:14–23. 10.1111/j.1600-0838.2010.01069.x 20149143

[26] Randers MB Petersen J Andersen LJ: Short-term street soccer improves fitness and cardiovascular health status of homeless men. *Eur J Appl Physiol.* 2012;112(6):2097–106. 10.1007/s00421-011-2171-1 21956486

[27] Quine S Kendig H Russell C: Health promotion for socially disadvantaged groups: The case of homeless older men in Australia. *Health Promot Int.* 2004;19(2):157–65. 10.1093/heapro/dah203 15128707

[28] Ballard FA: Homeless sheltered women's health promotion behaviors.University of North Carolina at Greensboro,2009. Reference Source

[29] Wilson M: Health-promoting behaviors of sheltered homeless women. *Fam Community Health.* 2005;28(1):51–63. 10.1097/00003727-200501000-00008 15625506

[30] Brown RT Kiely DK Bharel M: Geriatric syndromes in older homeless adults. *J Gen Intern Med.* 2012;27(1):16–22. 10.1007/s11606-011-1848-9 21879368 PMC3250555

[31] Raven MC Tieu L Lee CT: Emergency Department Use in a Cohort of Older Homeless Adults: Results From the HOPE HOME Study. *Acad Emerg Med.* 2017;24(1):63–74. 10.1111/acem.13070 27520382 PMC5857347

[32] Fried LP Tangen CM Walston J: Frailty in older adults: evidence for a phenotype. *J Gerontol A Biol Sci Med Sci.* 2001;56(3):M146–56. 10.1093/gerona/56.3.m146 11253156

[33] Marmolejo MA Medhanie M Tarleton HP: Musculoskeletal Flexibility and Quality of Life: A Feasibility Study of Homeless Young Adults in Los Angeles County. *Int J Exerc Sci.* 2018;11(4):968–79. 30147826 10.70252/PGMO3065 PMC6102196

[34] Haff G Triplett T: Essentials of Strength Training and Conditioning.National Strength and Conditioning Association Illinois, USA: Champaign;2016. Reference Source

[35] Lauretani F Russo CR Bandinelli S: Age-associated changes in skeletal muscles and their effect on mobility: an operational diagnosis of sarcopenia. *J Appl Physiol (1985).* 2003;95(5):1851–60. 10.1152/japplphysiol.00246.2003 14555665

[36] Patanwala M Tieu L Ponath C: Physical, Psychological, Social, and Existential Symptoms in Older Homeless-Experienced Adults: An Observational Study of the Hope Home Cohort. *J Gen Intern Med.* 2018;33(5):635–43. 10.1007/s11606-017-4229-1 29185174 PMC5910332

[37] Kroenke K Spitzer RL Williams JB: The PHQ-15: validity of a new measure for evaluating the severity of somatic symptoms. *Psychosom Med.* 2002;64(2):258–66. 10.1097/00006842-200203000-00008 11914441

[38] Ware JE Kosinski M Keller SD: How to score the SF-12 physical and mental health summary scales.Lincoln, RI: QualityMetric Incorporated;1998. Reference Source

[39] Gadermann AM Hubley AM Russell LB: Subjective healthrelated quality of life in homeless and vulnerably housed individuals and its relationship with self-reported physical and mental health status. *Soc Indic Res.* 2014;116(2):341–52. 10.1007/s11205-013-0302-2

[40] Bazari A Patanwala M Kaplan LM: *'The Thing that Really Gets Me Is the Future'*: Symptomatology in Older Homeless Adults in the HOPE HOME Study. *J Pain Symptom Manage.* 2018;56(2):195–204. 10.1016/j.jpainsymman.2018.05.011 29783004 PMC6050110

[41] Kendzor DE Allicock M Businelle MS: Evaluation of a Shelter-Based Diet and Physical Activity Intervention for Homeless Adults. *J Phys Act Health.* 2017;14(2):88–97. 10.1123/jpah.2016-0343 27775471

[42] Courneya KS Bobick TW: Integrating the theory of planned behaviour with the processes and stages of change in the exercise domain. *Psychol Sport Exerc.* 2000;1:41–56. 10.1016/S1469-0292(00)00006-6

[43] Walker SN Sechrist K Pender N: The health-promoting lifestyle profile II. Omaha, University of Nebraska Medical Center, College of Nursing.1995.

[44] Nyamathi AM Leake B Gelberg L: Sheltered versus nonsheltered homeless women differences in health, behavior, victimization, and utilization of care. *J Gen Intern Med.* 2000;15(8):565–72. 10.1046/j.1525-1497.2000.07007.x 10940149 PMC1495574

[45] Randers MB Marschall J Nielsen TT: Heart rate and movement pattern in street soccer for homeless women. *German Journal of Exercise and Sport Research.* 2018;48:211–7. 10.1007/s12662-018-0503-6

[46] Stringer C Loosemore M Moller E: Promoting physical activity in vulnerable adults 'at risk' of homelessness: a randomised controlled trial protocol. *BMJ Open.* 2019;9(3):e026466. 10.1136/bmjopen-2018-026466 30904872 PMC6475440

[47] Guralnik JM Simonsick EM Ferrucci L: A short physical performance battery assessing lower extremity function: association with self-reported disability and prediction of mortality and nursing home admission. *J Gerontol.* 1994;49(2):M85–94. 10.1093/geronj/49.2.m85 8126356

[48] Ara I Aparicio-Ugarriza R Morales-Barco D: Physical activity assessment in the general population; validated self-report methods. *Nutr Hosp.* 2015;31 Suppl 3:211–8. 10.3305/nh.2015.31.sup3.8768 25719788

[49] Chalmers I Glasziou P: Systematic reviews and research waste. *Lancet.* 2016;387(10014):122–3. 10.1016/S0140-6736(15)01353-7 26841991

[50] Tadros A Layman SM Brewer MP: A 5-year comparison of ED visits by homeless and nonhomeless patients. *Am J Emerg Med.* 2016;34(5):805–8. 10.1016/j.ajem.2016.01.012 26935222

[51] Brown RT Kiely DK Bharel M: Factors associated with geriatric syndromes in older homeless adults. *J Health Care Poor Underserved.* 2013;24(2):456–68. 10.1353/hpu.2013.0077 23728022 PMC3671483

[52] Kelsey JL Berry SD Procter-Gray E: Indoor and outdoor falls in older adults are different: the maintenance of balance, independent living, intellect, and Zest in the Elderly of Boston Study. *J Am Geriatr Soc.* 2010;58(11):2135–41. 10.1111/j.1532-5415.2010.03062.x 20831726 PMC2975756

[53] Leveille SG Kiel DP Jones RN: The MOBILIZE Boston Study: design and methods of a prospective cohort study of novel risk factors for falls in an older population. *BMC Geriatr.* 2008;8:16. 10.1186/1471-2318-8-16 18638389 PMC2500010

[54] Pober DM Freedson PS Kline GM: Development and validation of a one-mile treadmill walk test to predict peak oxygen uptake in healthy adults ages 40 to 79 years. *Can J Appl Physiol.* 2002;27(6):575–89. 10.1139/h02-033 12500996

[55] Peters MD Godfrey CM Khalil H: Guidance for conducting systematic scoping reviews. *Int J Evid Based Healthc.* 2015;13(3):141–6. 10.1097/XEB.0000000000000050 26134548

[56] Williamson PR Altman DG Blazeby JM: Developing core outcome sets for clinical trials: issues to consider. *Trials.* 2012;13:132. 10.1186/1745-6215-13-132 22867278 PMC3472231

