# Supplementary material for: Physical functioning limitations and physical activity of people experiencing homelessness: A scoping review
Source: HRB Open Res. 2021 Mar 1;3:14. Originally published 2020 Apr 16. [Version 2] doi: 10.12688/hrbopenres.13011.2 (PMC7934094; doi:10.12688/hrbopenres.13011.2)
Supplement: Peer review history

# Peer review: Reviewer response for version 2

**DOI:** 10.21956/hrbopenres.14378.r29009

**Published:** 2021-03-04

**Authors:** Cilia Mejia-Lancheros

**Affiliations:** 1 MAP Centre for Urban Health Solutions, St Michael's Hospital, Li Ka Shing Knowledge Institute, Toronto, ON, Canada

I think the authors have done a great job in addressing the comments and suggestions; therefore, I do not have any further revisions to request. Congratulations to the authors.

Are the rationale for, and objectives of, the Systematic Review clearly stated?

Yes

Is the statistical analysis and its interpretation appropriate?

Not applicable

Are sufficient details of the methods and analysis provided to allow replication by others?

Partly

Are the conclusions drawn adequately supported by the results presented in the review?

Yes

Reviewer Expertise:

Social epidemiology and public health scientist with expertise on health inequalities, social determinants of health, homelessness, housing and health.

I confirm that I have read this submission and believe that I have an appropriate level of expertise to confirm that it is of an acceptable scientific standard.

# Peer review: Reviewer response for version 1

**DOI:** 10.21956/hrbopenres.14104.r28429

**Published:** 2020-12-07

**Authors:** Cilia Mejia-Lancheros

**Affiliations:** 1 MAP Centre for Urban Health Solutions, St Michael's Hospital, Li Ka Shing Knowledge Institute, Toronto, ON, Canada

Overview

 The study assessed the existing evidence on physical functioning limitation and physical activity among homeless adults using the scoping review framework. The findings highlight that people who experience homelessness have a high level of physical functioning impairment and low physical activities, which provides evidence of this population's low health status. It also shows the need for effective community-based and clinical-based interventions to diminish their premature physical functioning decline and poor health and social well-being.

 Overall, the paper is well-written and structured. However, I have some suggestions below that might strengthen the paper's quality, the interpretation of the findings and the derived knowledge translation process.

 Abstract

1. In the statement, "This review aimed to evaluate what is known about physical functional limitations and physical activity levels and how these constructs are measured in adults experiencing homelessness", I think physical functional limitations and physical activity are more conditions and abilities than constructs.
2. In the abstract methods: I suggest including the period within which the reviewed literature was considered.
3. In the statement "The following physical focused measures …" I suggest separating the specific outcomes related to physical functional limitations than those related to physical activity level by not putting all together in the same statement.

Introduction

1. The statistic of the figure of homelessness in the USA and Canada should be accompanied by the referring period. For example, for Canada, the figures refer to 2017 and for the USA to 2016.
2. By 'tri-morbidity, do you mean: mental illness, physical illness(included chronic diseases) and substance use disorders? Since chronic diseases are communally referred to as physical diseases. 
3. By housed individuals, do you mean people with stably housed or non-homeless people? I think it is essential to be more specific.
4. In the statement, "An abundance of epidemiological highlights physical inactivity as a significant predictor of cardiovascular disease, type 2 diabetes mellitus, obesity, some cancers, poor skeletal health, some aspects of mental health, and overall mortality, as well as poor quality of life" please specify the population.
5. The statement "Improved understanding of physical variables is important…" please specify what physical variable you are referring to. Perhaps, physical functioning and activity?
6. In the scoping review, it is important to specify that the study population is adults experiencing homelessness. As there are also youth and children who experience homelessness.

Methods

*Data sources and searches *

1. Who was that subject expert? An academic expert in homelessness? A person with lived experience of homelessness? Please, elaborate on it.
2. Please, report the period in which the research for studies was conducted. It will allow the replicability or update of the searching strategy.

*Physically focused definitions employed in this review  *

1. "We employed the definition of functional limitation" do you mean the definition of physical functioning? Please specify.

*Selection of studies*

1. I wondered if you used any strategy or tool to perform the appraisal of the included studies' quality. Please elaborate on this matter.

*Data analysis*

1. As you well-know, the scope of a scoping review is not to pool empirical findings using statistical methods as it happens in a systematic and meta-analysis review. Instead, it is more synthesized or summarized the findings. Thus, the statement "Due to the heterogeneity of study design, interventions and outcomes, a narrative synthesis was conducted" is no application for the scoping review.

Results

1. In the result, you describe female and male as one of the demographic characteristics of the revised studies' participants. In Table 2, you report this characteristic as gender. Gender (e.g. men/women) is more a social and identity construct, while sex (female/male) more a biological characteristic. Considering these differences, please clarify whether all studies measure gender or biological sex or both. If both, please include them as two distinct characteristics. This health to inform any potential gender-based or biological sex-based differences and similitudes, and gaps in the review' findings.
2. Perhaps briefly summarize the definition of homelessness employed in the included studies could give a more comprehensive view of what groups of homeless people were more likely to be studied.
3. Please state clearly in the results (text and Table 1) which of the findings you are presented as "Physical Focused Outcomes (measure)" are those referring to physical functioning measures only and which to the physical activity only. This is important as they are the two primary distinct outcomes assessed in your review. You may consider two add one column for the physical functioning outcome and the other for the physical activity outcomes. In Table 3, If the measures you are presented there are those regarding physical functioning, please state that clearly in the table's title and the variable level within the table.
4. As you included both qualitative and quantitative studies, it would better to present the characteristics and findings of those two groups of designed papers separately in the tables. Moreover, the number of studies with qualitative design reported in the results text do not concord with the number of qualitative studies present in Table 1.
5. Perhaps the synthesis of the studies' results could be organized under two main headings: Physical functioning and physical activity, as they are the two primary outcomes, you were assessed. Under these two main subheadings, you can summarise the related physical functional sub-outcomes and physical activity sub-outcomes as sub-headings.
6. In the results, perhaps before summarising the physical functioning and physical activity-related findings, presenting a brief description of the participants' comorbid profile could contribute to having a more comprehensive view of the health status of the study population. As many of those comorbid conditions are directly related to their physical functioning and physical activity status/levels.

Discussion

1. "…but the diversity of measures limited our ability to synthesize data for the purposes of this review." I think this part of the statement is unnecessary. As I may have said previously, a scoping review aims to present a broad view and synthesize of what has been studied, how it has been studied, identify gaps and give some recommendation on the reviewed topic, but not too pool results.
2. Please considering my previous comment regarding differences between gender and biological sex. Please discuss the findings accordingly.
3. Discuss the potential ethno-racial gaps in the studied topic.
4. Please extend the potential implications of the findings for more community-based support services and policy, especially community-based (health, housing, social) interventions that can improve this population's physical functioning and physical activities.

Figure 1:

1. Please state the reason for excluding records. You only included those included in the database searching. Are those figures also include the studies identified by searching grey literature?
2. There are discrepancies between the numbers of records found reported in Figure 1 and those reported in the result text. Please correct it accordingly.

Are the rationale for, and objectives of, the Systematic Review clearly stated?

Yes

Is the statistical analysis and its interpretation appropriate?

Not applicable

Are sufficient details of the methods and analysis provided to allow replication by others?

Partly

Are the conclusions drawn adequately supported by the results presented in the review?

Yes

Reviewer Expertise:

Social epidemiology and public health scientist with expertise on health inequalities, social determinants of health, homelessness, housing and health.

I confirm that I have read this submission and believe that I have an appropriate level of expertise to confirm that it is of an acceptable scientific standard, however I have significant reservations, as outlined above.

# Response

**Published:** 2021-01-26

**Authors:** Julie Broderick

**Affiliations:** Trinity College Dublin, Ireland

The authors would like to sincerely thank the reviewer for their extremely insightful comments. We have numbered each comment and responded to each in turn as per below. 

 Overview

 The study assessed the existing evidence on physical functioning limitation and physical activity among homeless adults using the scoping review framework. The findings highlight that people who experience homelessness have a high level of physical functioning impairment and low physical activities, which provides evidence of this population's low health status. It also shows the need for effective community-based and clinical-based interventions to diminish their premature physical functioning decline and poor health and social well-being.

 Overall, the paper is well-written and structured. However, I have some suggestions below that might strengthen the paper's quality, the interpretation of the findings and the derived knowledge translation process.

 Abstract

**R2 Comment 1**

 1.In the statement, "This review aimed to evaluate what is known about physical functional limitations and physical activity levels and how these constructs are measured in adults experiencing homelessness", I think physical functional limitations and physical activity are more conditions and abilities than constructs.

**Response to R2 Comment 1:** This has been changed to: ‘The aim of this scoping review was to evaluate what is known about physical functional limitations and physical activity levels and how they are measured in adults experiencing homelessness.’

**R2 Comment 2**

 2. In the abstract methods: I suggest including the period within which the reviewed literature was considered.

**Response to R2 Comment 2:** ‘From inception to 16.01.19’ has been added to the abstract

**R2 Comment 3**

 3. In the statement "The following physical focused measures …" I suggest separating the specific outcomes related to physical functional limitations than those related to physical activity level by not putting all together in the same statement. ****

**Response to R2 Comment 3:** This has been changed to; ‘The following outcomes related to physical functioning were reported; mobility levels (n=3), frailty (n=1), flexibility (n=2), strength (n=1), physical symptom burden (n=3), and exercise capacity (n=3). Eight studies reported outcomes related to physical activity.’

 Introduction

**R2 Comment 4**

 4.The statistic of the figure of homelessness in the USA and Canada should be accompanied by the referring period. For example, for Canada, the figures refer to 2017 and for the USA to 2016.

**Response to R2 Comment 4: **‘based on data from 2017, 2016 and 2017 respectively’ has been added in relating to this point.

**R2 Comment 5**

 5. By 'tri-morbidity, do you mean: mental illness, physical illness(included chronic diseases) and substance use disorders? Since chronic diseases are communally referred to as physical diseases. 

**Response to R2 Comment 5: **This has been changed to a ‘tri-morbidity’ of mental ill health, physical ill health, and addiction’

**R2 Comment 6**

 6. By housed individuals, do you mean people with stably housed or non-homeless people? I think it is essential to be more specific.

**Response to R2 Comment 6:** ‘Stably’ housed is inserted to clarify this point

**R2 Comment 7**

 7. In the statement, "An abundance of epidemiological highlights physical inactivity as a significant predictor of cardiovascular disease, type 2 diabetes mellitus, obesity, some cancers, poor skeletal health, some aspects of mental health, and overall mortality, as well as poor quality of life" please specify the population. ****

**Response to R2 Comment 7:** This point has been rewritten as follows; ‘The benefits of physical activity are well known and recent guidelines (1) have highlighted additional benefits of physical activity in terms of cognitive health health-related quality of life, mental health and sleep which has largely been explored in healthy populations. Information on physical activity levels among individuals who are homeless is not well known (2).

**R2 Comment 8**

 8. The statement "Improved understanding of physical variables is important…" please specify what physical variable you are referring to. Perhaps, physical functioning and activity?

**Response to R2 Comment 8:** ‘Improved understanding of physical functioning and physical activity’

**R2 Comment 9**

 9. In the scoping review, it is important to specify that the study population is adults experiencing homelessness. As there are also youth and children who experience homelessness

**Response to R2 Comment 9:** This has been specific as adults experiencing homelessness.

**Methods**

**Data sources and searches**

**R2 Comment 10**

 10.Who was that subject expert? An academic expert in homelessness? A person with lived experience of homelessness? Please, elaborate on it.

**Response to R2 Comment 10:** ‘The subject expert was a medical consultant who developed an inclusion health service for adults experiencing homelessness and is the clinical lead for service provision for people experiencing homelessness admitted to a large acute inner-city hospital in Dublin, Ireland’

**R2 Comment 11**

 11.Please, report the period in which the research for studies was conducted. It will allow the replicability or update of the searching strategy.

**Response to R2 Comment 11:** This was from inception to 16.01.19.

**R2 Comment 12**

 12. Physically focused definitions employed in this review  "We employed the definition of functional limitation" do you mean the definition of physical functioning? Please specify.

**Response to R2 Comment 12:** ‘We employed Nagi’s (3) definition of functional limitations as restrictions in the basic performance of the person such as limitations in the performance of locomotor tasks, such as the person’s gait, and basic mobility. Although not the specific focus of this review, factors that relate to physical functioning limitations such as, but not limited to, frailty, physical symptom burden and cardiovascular fitness were included if reported in studies sourced’

 Selection of studies

**R2 Comment 13**

 13.I wondered if you used any strategy or tool to perform the appraisal of the included studies' quality. Please elaborate on this matter.

**Response to R2 Comment 13:** We followed the Joanna Briggs Institute for Evidence Synthesis Scoping Review Chapter 11 ( https://wiki.jbi.global/display/MANUAL/Chapter+11%3A+Scoping+reviews). In this it states that formal assessment of methodological quality of the included sources of evidence of a scoping review is generally not performed, and was therefore not conducted as part of this scoping review.

**R2 Comment 14**

 14.As you well-know, the scope of a scoping review is not to pool empirical findings using statistical methods as it happens in a systematic and meta-analysis review. Instead, it is more synthesized or summarized the findings. Thus, the statement "Due to the heterogeneity of study design, interventions and outcomes, a narrative synthesis was conducted" is no application for the scoping review.

**Response to R2 Comment 14:** We apologise for this omission, this point has been removed.

**R2 Comment 15**

 15.In the result, you describe female and male as one of the demographic characteristics of the revised studies' participants. In Table 2, you report this characteristic as gender. Gender (e.g. men/women) is more a social and identity construct, while sex (female/male) more a biological characteristic. Considering these differences, please clarify whether all studies measure gender or biological sex or both. If both, please include them as two distinct characteristics. This health to inform any potential gender-based or biological sex-based differences and similitudes, and gaps in the review' findings.

**Response to R2 Comment 15: **This refers to biological sex rather than gender and has been changed in the table and text.

**R2 Comment 16**

 16. Perhaps briefly summarize the definition of homelessness employed in the included studies could give a more comprehensive view of what groups of homeless people were more likely to be studied.

**Response to R2 Comment 16:** To clarify this information the column ‘Living arrangement’ in table 1 was changed to ‘Definition of homelessness/living arrangement/access to services’ changed to ‘definition of homelessness’. The following was added to the results section ‘A formal operational definition of homeless was included in one study only (4). The living arrangement of participants was outlined in the recruitment strategy and/or eligibility criteria of remaining studies. The majority of studies included participants in shelter accommodation.’

**R2 Comment 17**

 17.Please state clearly in the results (text and Table 1) which of the findings you are presented as "Physical Focused Outcomes (measure)" are those referring to physical functioning measures only and which to the physical activity only. This is important as they are the two primary distinct outcomes assessed in your review. You may consider two add one column for the physical functioning outcome and the other for the physical activity outcomes. In Table 3, If the measures you are presented there are those regarding physical functioning, please state that clearly in the table's title and the variable level within the table.

**Response to R2 Comment 17:** An additional column has been added to Table 1  ‘Physical functioning measure or Physical activity measure’

**R2 Comment 18**

 18.As you included both qualitative and quantitative studies, it would better to present the characteristics and findings of those two groups of designed papers separately in the tables. Moreover, the number of studies with qualitative design reported in the results text do not concord with the number of qualitative studies present in Table 1.

**Response to R2 Comment 18:** A sub heading to differentiate between qualitative and quantitative studiers is now included in Tables 1 and 2. The discrepancy between the number of qualitative studies in the text and tables has been amended.

**R2 Comment 19**

 19.Perhaps the synthesis of the studies' results could be organized under two main headings: Physical functioning and physical activity, as they are the two primary outcomes, you were assessed. Under these two main subheadings, you can summarise the related physical functional sub-outcomes and physical activity sub-outcomes as sub-headings.

**Response to R2 Comment 19:** These sub-headings have been inserted into the results section.

**R2 Comment 20**

  In the results, perhaps before summarising the physical functioning and physical activity related findings, presenting a brief description of the participants' comorbid profile could contribute to having a more comprehensive view of the health status of the study population. As many of those comorbid conditions are directly related to their physical functioning and physical activity status/levels.

**Response to R2 Comment 20:** The following has been added to the results section ‘Despite the relatively low mean/median age of participants [2 ^nd^ decade (n=2 studies), 3 ^rd^ decade (n=2 studies), 4 ^th^ decade (n=5 studies), 50 ^th^ decade (n=5 studies), 60th decade, (n=2 studies), participants experienced a high burden of physical and mental conditions. From data presented in included studies, rates of hypertension ranged from 20.4% to 59%, arthritis from 16.8% to 46.8%, diabetes from 14% to 18.3%  and depression from 34% to 59.6%.’

 Discussion

**R2 Comment 21**

 "…but the diversity of measures limited our ability to synthesize data for the purposes of this review." I think this part of the statement is unnecessary. As I may have said previously, a scoping review aims to present a broad view and synthesize of what has been studied, how it has been studied, identify gaps and give some recommendation on the reviewed topic, but not too pool results.

**Response to R2 Comment 21:** This has been addressed above in R2 comment 14.

**R2 Comment 22**

 22.Please considering my previous comment regarding differences between gender and biological sex. Please discuss the findings accordingly.

**Response to R2 Comment 22:** ‘This review included 2,018 participants, of which females were underrepresented as over 70% of review participants were male. This reflects that 4 studies exclusively included males, whereas only 2 studies only included females, and relatively there was a higher proportion of males than females in the remaining studies. Less therefore appears to be known about the physical profile of females experiencing homelessness compared to males. Sex as a biological characteristic was reported in studies was reported rather than gender which is more a social and identity construct (5). Is is known that transgender people are disproportionally represented among homeless populations (6) but this group were not represented in studies included in this review.

**R2 Comment 23**

 23.Discuss the potential ethno-racial gaps in the studied topic.

**Response to R2 Comment 23:**  ‘In the US based studies 59.6% of participants were African American, while a lower proportion were white (29.8%). This reflects the high proportion of African Americans among homeless populations in the US (7). Indigenous people are also over-represented among homeless populations (8) which likely mirrors the proportion of Aboriginal people in a Canadian study (9) included in this review. It is possible that in other studies this group may have been under-represented or not specifically reported. Out of 10 US based studies, one reported the proportion of American Indian participants was 4.8%, and another quoted that 10% of participants were American Indian/Alaska Natives/Native Hawaiian/Pacific Islanders. Most of the rest of the studies included categories of ‘other’ in which it was likely native populations were subsumed. Similarly, there may have been an under-representation of Latino people and people of mixed race heritage but absolute proportions of different ethnic groups among homeless populations are likely to be context specific.’

**R2 Comment 24**

 24.Please extend the potential implications of the findings for more community-based support services and policy, especially community-based (health, housing, social) interventions that can improve this population's physical functioning and physical activities.

**R2 Comment 24:** In relation to this point we have added in the following ‘As all studies included in this review were community based, the generally low level of physical functioning and physical activity of this population is relevant to a broad spectrum of community based services including housing, social health services. Housing services should bear accessibility in mind and social activities should incorporate a physical/exercise component where possible. ‘

 Figure 1:

**R2 Comment 25**

 25.Please state the reason for excluding records. You only included those included in the database searching. Are those figures also include the studies identified by searching grey literature?

**Response to R2 Comment 25:** Records were excluded as they did not report outcomes related to physical functioning ad physical activity in adults who were homeless.

**R2 Comment 26**

 26.There are discrepancies between the numbers of records found reported in Figure 1 and those reported in the result text. Please correct it accordingly.

**Response to R2 Comment 26:** The discrepancies have been amended.

# Response

**Published:** 2021-02-19

**Authors:** Julie Broderick

**Affiliations:** Trinity College Dublin, Ireland

**Dear Reviewer 2,**

**Thank you for your very insightful comments, in response to your comments; we have made the following changes to the review.**

**Each comment is listed, numbered, and responded to in turn.**

**Kind Regards, Julie Broderick and co-authors**

**From R2** Overview

 The study assessed the existing evidence on physical functioning limitation and physical activity among homeless adults using the scoping review framework. The findings highlight that people who experience homelessness have a high level of physical functioning impairment and low physical activities, which provides evidence of this population's low health status. It also shows the need for effective community-based and clinical-based interventions to diminish their premature physical functioning decline and poor health and social well-being.

 Overall, the paper is well-written and structured. However, I have some suggestions below that might strengthen the paper's quality, the interpretation of the findings and the derived knowledge translation process.

 Abstract

**R2 Comment 1**

 1.In the statement, "This review aimed to evaluate what is known about physical functional limitations and physical activity levels and how these constructs are measured in adults experiencing homelessness", I think physical functional limitations and physical activity are more conditions and abilities than constructs.

**Response to R2 Comment 1:** This has been changed to: ‘The aim of this scoping review was to evaluate what is known about physical functional limitations and physical activity levels and how they are measured in adults experiencing homelessness.’

**R2 Comment 2**

 2. In the abstract methods: I suggest including the period within which the reviewed literature was considered.

**Response to R2 Comment 2:** ‘From inception to 16.01.19’ has been added to the abstract

**R2 Comment 3**

 3. In the statement "The following physical focused measures …" I suggest separating the specific outcomes related to physical functional limitations than those related to physical activity level by not putting all together in the same statement. ****

**Response to R2 Comment 3:** This has been changed to; ‘The following outcomes related to physical functioning were reported; mobility levels (n=3), frailty (n=1), flexibility (n=2), strength (n=1), physical symptom burden (n=3), and exercise capacity (n=3). Eight studies reported outcomes related to physical activity.’

 Introduction

**R2 Comment 4**

 4.The statistic of the figure of homelessness in the USA and Canada should be accompanied by the referring period. For example, for Canada, the figures refer to 2017 and for the USA to 2016.

**Response to R2 Comment 4: **‘based on data from 2017, 2016 and 2017 respectively’ has been added in relating to this point.

**R2 Comment 5**

 5. By 'tri-morbidity, do you mean: mental illness, physical illness(included chronic diseases) and substance use disorders? Since chronic diseases are communally referred to as physical diseases. 

**Response to R2 Comment 5: **This has been changed to a ‘tri-morbidity’ of mental ill health, physical ill health, and addiction’

**R2 Comment 6**

 6. By housed individuals, do you mean people with stably housed or non-homeless people? I think it is essential to be more specific.

**Response to R2 Comment 6:** ‘Stably’ housed is inserted to clarify this point

**R2 Comment 7**

 7. In the statement, "An abundance of epidemiological highlights physical inactivity as a significant predictor of cardiovascular disease, type 2 diabetes mellitus, obesity, some cancers, poor skeletal health, some aspects of mental health, and overall mortality, as well as poor quality of life" please specify the population. ****

**Response to R2 Comment 7:** This point has been rewritten as follows; ‘The benefits of physical activity are well known and recent guidelines (1) have highlighted additional benefits of physical activity in terms of cognitive health health-related quality of life, mental health and sleep which has largely been explored in healthy populations. Information on physical activity levels among individuals who are homeless is not well known (2).

**R2 Comment 8**

 8. The statement "Improved understanding of physical variables is important…" please specify what physical variable you are referring to. Perhaps, physical functioning and activity?

**Response to R2 Comment 8:** ‘Improved understanding of physical functioning and physical activity’

**R2 Comment 9**

 9. In the scoping review, it is important to specify that the study population is adults experiencing homelessness. As there are also youth and children who experience homelessness

**Response to R2 Comment 9:** This has been specific as adults experiencing homelessness.

**Methods**

**Data sources and searches**

**R2 Comment 10**

 10.Who was that subject expert? An academic expert in homelessness? A person with lived experience of homelessness? Please, elaborate on it.

**Response to R2 Comment 10:** ‘The subject expert was a medical consultant who developed an inclusion health service for adults experiencing homelessness and is the clinical lead for service provision for people experiencing homelessness admitted to a large acute inner-city hospital in Dublin, Ireland’

**R2 Comment 11**

 11.Please, report the period in which the research for studies was conducted. It will allow the replicability or update of the searching strategy.

**Response to R2 Comment 11:** This was from inception to 16.01.19.

**R2 Comment 12**

 12. Physically focused definitions employed in this review  "We employed the definition of functional limitation" do you mean the definition of physical functioning? Please specify.

**Response to R2 Comment 12:** ‘We employed Nagi’s (3) definition of functional limitations as restrictions in the basic performance of the person such as limitations in the performance of locomotor tasks, such as the person’s gait, and basic mobility. Although not the specific focus of this review, factors that relate to physical functioning limitations such as, but not limited to, frailty, physical symptom burden and cardiovascular fitness were included if reported in studies sourced’

 Selection of studies

**R2 Comment 13**

 13.I wondered if you used any strategy or tool to perform the appraisal of the included studies' quality. Please elaborate on this matter.

**Response to R2 Comment 13:** We followed the Joanna Briggs Institute for Evidence Synthesis Scoping Review Chapter 11 ( https://wiki.jbi.global/display/MANUAL/Chapter+11%3A+Scoping+reviews). In this it states that formal assessment of methodological quality of the included sources of evidence of a scoping review is generally not performed, and was therefore not conducted as part of this scoping review.

**R2 Comment 14**

 14.As you well-know, the scope of a scoping review is not to pool empirical findings using statistical methods as it happens in a systematic and meta-analysis review. Instead, it is more synthesized or summarized the findings. Thus, the statement "Due to the heterogeneity of study design, interventions and outcomes, a narrative synthesis was conducted" is no application for the scoping review.

**Response to R2 Comment 14:** We apologise for this omission, this point has been removed.

**R2 Comment 15**

 15.In the result, you describe female and male as one of the demographic characteristics of the revised studies' participants. In Table 2, you report this characteristic as gender. Gender (e.g. men/women) is more a social and identity construct, while sex (female/male) more a biological characteristic. Considering these differences, please clarify whether all studies measure gender or biological sex or both. If both, please include them as two distinct characteristics. This health to inform any potential gender-based or biological sex-based differences and similitudes, and gaps in the review' findings.

**Response to R2 Comment 15: **This refers to biological sex rather than gender and has been changed in the table and text.

**R2 Comment 16**

 16. Perhaps briefly summarize the definition of homelessness employed in the included studies could give a more comprehensive view of what groups of homeless people were more likely to be studied.

**Response to R2 Comment 16:** To clarify this information the column ‘Living arrangement’ in table 1 was changed to ‘Definition of homelessness/living arrangement/access to services’ changed to ‘definition of homelessness’. The following was added to the results section ‘A formal operational definition of homeless was included in one study only (4). The living arrangement of participants was outlined in the recruitment strategy and/or eligibility criteria of remaining studies. The majority of studies included participants in shelter accommodation.’

**R2 Comment 17**

 17.Please state clearly in the results (text and Table 1) which of the findings you are presented as "Physical Focused Outcomes (measure)" are those referring to physical functioning measures only and which to the physical activity only. This is important as they are the two primary distinct outcomes assessed in your review. You may consider two add one column for the physical functioning outcome and the other for the physical activity outcomes. In Table 3, If the measures you are presented there are those regarding physical functioning, please state that clearly in the table's title and the variable level within the table.

**Response to R2 Comment 17:** An additional column has been added to Table 1  ‘Physical functioning measure or Physical activity measure’

**R2 Comment 18**

 18.As you included both qualitative and quantitative studies, it would better to present the characteristics and findings of those two groups of designed papers separately in the tables. Moreover, the number of studies with qualitative design reported in the results text do not concord with the number of qualitative studies present in Table 1.

**Response to R2 Comment 18:** A sub heading to differentiate between qualitative and quantitative studiers is now included in Tables 1 and 2. The discrepancy between the number of qualitative studies in the text and tables has been amended.

**R2 Comment 19**

 19.Perhaps the synthesis of the studies' results could be organized under two main headings: Physical functioning and physical activity, as they are the two primary outcomes, you were assessed. Under these two main subheadings, you can summarise the related physical functional sub-outcomes and physical activity sub-outcomes as sub-headings.

**Response to R2 Comment 19:** These sub-headings have been inserted into the results section.

**R2 Comment 20**

  In the results, perhaps before summarising the physical functioning and physical activity related findings, presenting a brief description of the participants' comorbid profile could contribute to having a more comprehensive view of the health status of the study population. As many of those comorbid conditions are directly related to their physical functioning and physical activity status/levels.

**Response to R2 Comment 20:** The following has been added to the results section ‘Despite the relatively low mean/median age of participants [2 ^nd^ decade (n=2 studies), 3 ^rd^ decade (n=2 studies), 4 ^th^ decade (n=5 studies), 50 ^th^ decade (n=5 studies), 60th decade, (n=2 studies), participants experienced a high burden of physical and mental conditions. From data presented in included studies, rates of hypertension ranged from 20.4% to 59%, arthritis from 16.8% to 46.8%, diabetes from 14% to 18.3%  and depression from 34% to 59.6%.’

 Discussion

**R2 Comment 21**

 "…but the diversity of measures limited our ability to synthesize data for the purposes of this review." I think this part of the statement is unnecessary. As I may have said previously, a scoping review aims to present a broad view and synthesize of what has been studied, how it has been studied, identify gaps and give some recommendation on the reviewed topic, but not too pool results.

**Response to R2 Comment 21:** This has been addressed above in R2 comment 14.

**R2 Comment 22**

 22.Please considering my previous comment regarding differences between gender and biological sex. Please discuss the findings accordingly.

**Response to R2 Comment 22:** ‘This review included 2,018 participants, of which females were underrepresented as over 70% of review participants were male. This reflects that 4 studies exclusively included males, whereas only 2 studies only included females, and relatively there was a higher proportion of males than females in the remaining studies. Less therefore appears to be known about the physical profile of females experiencing homelessness compared to males. Sex as a biological characteristic was reported in studies was reported rather than gender which is more a social and identity construct (5). Is is known that transgender people are disproportionally represented among homeless populations (6) but this group were not represented in studies included in this review.

**R2 Comment 23**

 23.Discuss the potential ethno-racial gaps in the studied topic.

**Response to R2 Comment 23:**  ‘In the US based studies 59.6% of participants were African American, while a lower proportion were white (29.8%). This reflects the high proportion of African Americans among homeless populations in the US (7). Indigenous people are also over-represented among homeless populations (8) which likely mirrors the proportion of Aboriginal people in a Canadian study (9) included in this review. It is possible that in other studies this group may have been under-represented or not specifically reported. Out of 10 US based studies, one reported the proportion of American Indian participants was 4.8%, and another quoted that 10% of participants were American Indian/Alaska Natives/Native Hawaiian/Pacific Islanders. Most of the rest of the studies included categories of ‘other’ in which it was likely native populations were subsumed. Similarly, there may have been an under-representation of Latino people and people of mixed race heritage but absolute proportions of different ethnic groups among homeless populations are likely to be context specific.’

**R2 Comment 24**

 24.Please extend the potential implications of the findings for more community-based support services and policy, especially community-based (health, housing, social) interventions that can improve this population's physical functioning and physical activities.

**R2 Comment 24:** In relation to this point we have added in the following ‘As all studies included in this review were community based, the generally low level of physical functioning and physical activity of this population is relevant to a broad spectrum of community based services including housing, social health services. Housing services should bear accessibility in mind and social activities should incorporate a physical/exercise component where possible. ‘

 Figure 1:

**R2 Comment 25**

 25.Please state the reason for excluding records. You only included those included in the database searching. Are those figures also include the studies identified by searching grey literature?

**Response to R2 Comment 25:** Records were excluded as they did not report outcomes related to physical functioning ad physical activity in adults who were homeless.

**R2 Comment 26**

 26.There are discrepancies between the numbers of records found reported in Figure 1 and those reported in the result text. Please correct it accordingly.

**Response to R2 Comment 26:** The discrepancies have been amended.

 1.            Ding D, Mutrie N, Bauman A, Pratt M, Hallal PRC, Powell KE. Physical activity guidelines 2020: comprehensive and inclusive recommendations to activate populations. Lancet (London, England). 2020;396(10265):1780-2.

 2.            Taylor A, Murillo R, Businelle MS, Chen TA, Kendzor DE, McNeill LH, et al. Physical activity and sleep problems in homeless adults. PloS one. 2019;14(7):e0218870.

 3.            Nagi SZ. A STUDY IN THE EVALUATION OF DISABILITY AND REHABILITATION POTENTIAL: CONCEPTS, METHODS, AND PROCEDURES. Am J Public Health Nations Health. 1964;54(9):1568-79.

 4.            Chau S, Chin M, Chang J, Luecha A, Cheng E, Schlesinger J, et al. Cancer risk behaviors and screening rates among homeless adults in Los Angeles County. Cancer epidemiology, biomarkers & prevention : a publication of the American Association for Cancer Research, cosponsored by the American Society of Preventive Oncology. 2002;11(5):431-8.

 5.            Broughton DE, Brannigan RE, Omurtag KR. Sex and gender: you should know the difference. Fertility and sterility. 2017;107(6):1294-5.

 6.            Douglass KM, Polcari A, Najjar N, Kronenfeld J, Deshpande AR. Health Care for the Homeless Transgender Community: Psychiatric Services and Transition Care at a Student-Run Clinic. Journal of health care for the poor and underserved. 2018;29(3):940-8.

 7.            Wadhera RK, Khatana SAM, Choi E, Jiang G, Shen C, Yeh RW, et al. Disparities in Care and Mortality Among Homeless Adults Hospitalized for Cardiovascular Conditions. JAMA internal medicine. 2020;180(3):357-66.

 8.            Bingham B, Moniruzzaman A, Patterson M, Distasio J, Sareen J, O'Neil J, et al. Indigenous and non-Indigenous people experiencing homelessness and mental illness in two Canadian cities: A retrospective analysis and implications for culturally informed action. BMJ open. 2019;9(4):e024748.

 9.            Gadermann AM, Hubley, A.M., Russell, L.B. and Palepu, A. Subjective healthrelated quality of life in homeless and vulnerably housed individuals and its relationship

 with self-reported physical and mental health status. Social indicators research. 2014;116(2):341-52.

# Peer review: Reviewer response for version 1

**DOI:** 10.21956/hrbopenres.14104.r27323

**Published:** 2020-05-01

**Authors:** Jo Dawes

**Affiliations:** 1 Faculty of Health, Social Care and Education, a joint faculty between Kingston University and St George’s, University of London, London, UK; 2 Collaborative Centre for Inclusion Health, Department of Epidemiology and Public Health, University College London, London, UK

**Introduction**

 The background and rationale for this scoping review was clearly described and explains the importance of it being undertaken. The authors do address the aims of the paper, but are not explicit about the objectives (although objectives are mentioned later under “inclusion/ exclusion criteria”, so it would be useful to state these in introduction). The authors have rightly based their review on PCC (Populations, Concept, Context) rather than PICO (Participants, Interventions, Comparators, and Outcomes) as this is not a review of interventions. Their reasoning for this is adequately described.

**Methods**

 Much of the methods are well described. The authors are clear that this is a scoping review and have described using appropriate frameworks and guidance to inform their procedures. The authors are clear about information sources, for example language, databases searched and inclusion of grey literature. They noted that a subject expert was consulted, but did the authors also contact study authors to identify further literature? Although no date restriction was applied in the search, it would be useful to for authors to state the date of the last search. The current information given, it is not sufficiently detailed to be repeatable, for example, the authors could provide more detail of their search strategy, including limits used. Under “Physical focused definitions employed in the review” the authors provide definitions for functional limitation and physical activity. However, they also provide examples of functional limitations, but not physical activity, as physical activity is so broad, to add examples of search words used would add clarity.

 The PRISMA diagram is helpful, though it would be more accurate to describe Figure 1 as “PRISMA flow diagram of *selection for* review”, as it covers the selection/ inclusion process, not the whole review process. It might be more appropriate to make reference to Fig 1 earlier in “selection of studies” section. There was limited description of “data extraction” (it would be useful to hear more about what the specifically designed data extraction sheet contained). Again, limited information provided about data analysis - more detail on what was involved in the narrative synthesis would give greater transparency for the reader.

**Results**

 It is shown (in Fig 1) and described (in text) how many studies were excluded at each stage, but apart from the removal of duplicates, reasons for exclusions are not described. Also, the number of studies identified after duplicates removed does not match what is stated in text/Figure 1. (n=2832 in text/ n=2833 in Fig 1). For clarity, it might help to add *title and abstracts of* to the box “Records screened (n=1815)” in Figure 1.

 Authors have clearly expressed study characteristics in Table 1. However, it is stated in text they identified 11 quantitative studies and 4 qualitative studies, whereas only 3 studies are explicitly described as qualitative in Table 1. Was it the pilot study by Kendzor *et al. *(2015) which was also qualitative? If so, it would be useful if the authors made this clear. In Table 1, it would also be useful to be clearer about which studies addressed physical function, which addressed physical activity and which addressed both. For example, where Table 4 describes studies using physical activity measures, Gregg and Bedard (2016) and Marmolejo *et al.* (2018) feature, but in Table 1 the physical focused outcomes column does not clearly describe physical activity measures (for Gregg and Bedard (2016) exercise and intention to exercise are stated, but is not a measure of actual physical activity levels and Marmolejo *et al.* (2018) only a flexibility measure is described).

 Only minor, but in Table 2, there is slight inconsistency in presentation of data in “gender” column. Sometimes % and numbers, but sometimes only %, is this because numbers were not always provided in studies? If so, you could use “NS”, like you have in other parts of that table. Also, there are typos on p 8, para 1, lines 2-3: “many people homeless experiencing homelessness” and p 10, para 2, line 4: in this study is was”- replace “is” with “ *it*”.

 Due to the authors not formally assessing methodological quality of the studies, they were limited in how well they could acknowledge biases across studies.

**Discussion and Conclusion**

 A clear and succinct summary of the main findings and their implications for understanding the knowledge base, clinical practice and future research. Strengths and limitations of the paper appropriately identified and described.

Are the rationale for, and objectives of, the Systematic Review clearly stated?

Yes

Is the statistical analysis and its interpretation appropriate?

Not applicable

Are sufficient details of the methods and analysis provided to allow replication by others?

Partly

Are the conclusions drawn adequately supported by the results presented in the review?

Yes

Reviewer Expertise:

My area of expertise is physiotherapy education and access to healthcare amongst homeless and excluded populations.

I confirm that I have read this submission and believe that I have an appropriate level of expertise to confirm that it is of an acceptable scientific standard.

# Response

**Published:** 2021-01-26

**Authors:** Julie Broderick

**Affiliations:** Trinity College Dublin, Ireland

**The authors would like to sincerely thank the reviewer for their extremely insightful comments. For clarity we numbered each comment and responded to it in turn.**

**Introduction**

**R1 Comment 1**

 The background and rationale for this scoping review was clearly described and explains the importance of it being undertaken. The authors do address the aims of the paper, but are not explicit about the objectives (although objectives are mentioned later under “inclusion/ exclusion criteria”, so it would be useful to state these in introduction). The authors have rightly based their review on PCC (Populations, Concept, Context) rather than PICO (Participants, Interventions, Comparators, and Outcomes) as this is not a review of interventions. Their reasoning for this is adequately described.

**Response to R1 Comment 1: **The following has been added to the introduction section: **‘**Specific objectives were to evaluate the quantitative and qualitative literature on the following topics (i) physical functioning in adults experiencing homelessness, (ii) physical activity in adults experiencing homelessness, (iii) related secondary outcome measures such as frailty and cardiovascular fitness. In addition a further objective was added (iv) to evaluate measurement methods of physical outcomes in included studies.’

**Methods**

**R1 Comment 2**

 2. Much of the methods are well described. The authors are clear that this is a scoping review and have described using appropriate frameworks and guidance to inform their procedures. The authors are clear about information sources, for example language, databases searched and inclusion of grey literature. They noted that a subject expert was consulted, but did the authors also contact study authors to identify further literature?

**Response to R1 comment 2: **Authors did not contact study authors to identify further information. From the Joanna Briggs institute guidance for scoping reviews which we followed, this is not considered a mandatory step and was therefore not conducted as part of this review.

**R1 Comment 3**

 3. Although no date restriction was applied in the search, it would be useful to for authors to state the date of the last search.

**Response to R1 Comment 3:** The following has been included in the methods section: ‘from inception to 16.01.19.’

**R1 Comment 4**

 4. The current information given, it is not sufficiently detailed to be repeatable, for example, the authors could provide more detail of their search strategy, including limits used. Under “Physical focused definitions employed in the review” the authors provide definitions for functional limitation and physical activity. However, they also provide examples of functional limitations, but not physical activity, as physical activity is so broad, to add examples of search words used would add clarity.

**R1 Response to comment 4: **Precise details of both searches are included in Supplementary File 2. In relation to this point, the following has been added in the methods section:

 ‘Physical activity was considered any type of physical training or movement including any form of exercise, physical fitness or therapeutic movement. The full search strategy is available in Supplementary File 2.’

**R1 Comment 5**

 5.The PRISMA diagram is helpful, though it would be more accurate to describe Figure 1 as “PRISMA flow diagram of selection for review”, as it covers the selection/ inclusion process, not the whole review process.

**R1 Response to comment 5: **This has now been labelled as suggested.

**R1 Comment 6**

 6. It might be more appropriate to make reference to Fig 1 earlier in “selection of studies” section.

**R1 Response to comment 6: **Following the scoping review methodology from the Joanna Briggs Institute, Fig. 1 was referenced at the start of the results section. 

**R1 Comment 7**

 7. There was limited description of “data extraction” (it would be useful to hear more about what the specifically designed data extraction sheet contained).

**R1 Response to comment 7: **The following has been added to the methods section: ‘The data extraction instrument collected the following data relating to included studies (author, year of publication, country of study origin, inclusion criteria, living arrangements, physical focussed outcomes measured, participant characteristics (number of participants, age, gender, race/ethnicity, percentage with less than high school education, co-morbid conditions), physical focussed variables (physical variable measured, type of measure, total number of studies, authors, results), physical activity/sedentary behaviour focused measures (author, type of measure, measure subscale, main results). ‘

**R1 Comment 8**

 Again, limited information provided about data analysis - more detail on what was involved in the narrative synthesis would give greater transparency for the reader.

**Response to R1 Comment 8:** As per point from R2 Comment 19, synthesis of the studies' results was organized under two main headings: Physical functioning and physical activity. As per R1 comment 7 above, details of the data extraction too have also been provided.

**Results**

**R1 Comment 9**

 9. It is shown (in Fig 1) and described (in text) how many studies were excluded at each stage, but apart from the removal of duplicates, reasons for exclusions are not described.

**R1 Response to comment 9:** The reason for exclusion was not containing data relevant to physical functioning limitations or physical activity specific to adults who were homeless. The following has been added to the results section ’After excluding studies which did not containing data relevant to physical functioning limitations or physical activity specific to adults who were homeless, a total of 15 studies were deemed eligible for inclusion in this review.’

**R1** **Comment 10**

 10. Also, the number of studies identified after duplicates removed does not match what is stated in text/Figure 1. (n=2832 in text/ n=2833 in Fig 1).

**R1 Response to comment 10:** The correct value of 2833 is now included in the text.

**R1 Comment 11**

 11. For clarity, it might help to add title and abstracts of to the box “Records screened (n=1815)” in Figure 1.

**Response to comment R1 11:** This has now been inserted into Figure 1.

**R1 Comment 12**

 12. Authors have clearly expressed study characteristics in Table 1. However, it is stated in text they identified 11 quantitative studies and 4 qualitative studies, whereas only 3 studies are explicitly described as qualitative in Table 1. Was it the pilot study by Kendzor et al. (2015) which was also qualitative? If so, it would be useful if the authors made this clear.

**R1** **Response to comment 12:** Apologies for the lack of clarity around this. Two studies were qualitative (Bazari and Quine) and Table 1 has divided studies into quantitative and qualitative.

**R1** **Comment 13**

 13. In Table 1, it would also be useful to be clearer about which studies addressed physical function, which addressed physical activity and which addressed both. For example, where Table 4 describes studies using physical activity measures, Gregg and Bedard (2016) and Marmolejo et al. (2018) feature, but in Table 1 the physical focused outcomes column does not clearly describe physical activity measures (for Gregg and Bedard (2016) exercise and intention to exercise are stated, but is not a measure of actual physical activity levels and Marmolejo et al. (2018) only a flexibility measure is described).

**Response to R1 comment 13:** To improve clarity, a column ‘Physical-functioning measure or Physical activity ****measure’ construct measured has now been added to Table 1. Table 3 now includes Physical Functioning measures included in the review and Table 4 includes Physical Activity measures included in the review.

**R1 Comment 14**

 14 **. **Only minor, but in Table 2, there is slight inconsistency in presentation of data in “gender” column. Sometimes % and numbers, but sometimes only %, is this because numbers were not always provided in studies? If so, you could use “NS”, like you have in other parts of that table.

**Response to R1 comment 14:** NS has been added as suggested.

**R1 Comment 15**

 15. Also, there are typos on p 8, para 1, lines 2-3: “many people homeless experiencing homelessness” and p 10, para 2, line 4: in this study is was”- replace “is” with “it”. Due to the authors not formally assessing methodological quality of the studies, they were limited in how well they could acknowledge biases across studies.

**Response to R1 comment 15**: Typos have been amended. This is correct regarding inability to acknowledge biases across studies. Biases were not formally assessed based on Joanna Briggs methodology for scoping studies which was followed in this review.

 Discussion and Conclusion

**R1 Comment 16**

 16. A clear and succinct summary of the main findings and their implications for understanding the knowledge base, clinical practice and future research. Strengths and limitations of the paper appropriately identified and described.

**Response to comment R1 16:** Thank you to Reviewer 1 for these comments.

# Response

**Published:** 2021-02-19

**Authors:** Julie Broderick

**Affiliations:** Trinity College Dublin, Ireland

**Dear Reviewer 1,**

**Thank you for your very insightful comments, in response to your comments; we have made the following changes to the review.**

**Each comment is listed, numbered, and responded to in turn.**

**Kind Regards, Julie Broderick and co-authors**

**Introduction**

**R1 Comment 1**

 The background and rationale for this scoping review was clearly described and explains the importance of it being undertaken. The authors do address the aims of the paper, but are not explicit about the objectives (although objectives are mentioned later under “inclusion/ exclusion criteria”, so it would be useful to state these in introduction). The authors have rightly based their review on PCC (Populations, Concept, Context) rather than PICO (Participants, Interventions, Comparators, and Outcomes) as this is not a review of interventions. Their reasoning for this is adequately described.

**Response to R1 Comment 1: **The following has been added to the introduction section: **‘**Specific objectives were to evaluate the quantitative and qualitative literature on the following topics (i) physical functioning in adults experiencing homelessness, (ii) physical activity in adults experiencing homelessness, (iii) related secondary outcome measures such as frailty and cardiovascular fitness. In addition a further objective was added (iv) to evaluate measurement methods of physical outcomes in included studies.’

**Methods**

**R1 Comment 2**

 2. Much of the methods are well described. The authors are clear that this is a scoping review and have described using appropriate frameworks and guidance to inform their procedures. The authors are clear about information sources, for example language, databases searched and inclusion of grey literature. They noted that a subject expert was consulted, but did the authors also contact study authors to identify further literature?

**Response to R1 comment 2: **Authors did not contact study authors to identify further information. From the Joanna Briggs Institute Guidance for Scoping Reviews which we followed, this is not considered a mandatory step and was therefore not conducted as part of this review.

**R1 Comment 3**

 3. Although no date restriction was applied in the search, it would be useful to for authors to state the date of the last search.

**Response to R1 Comment 3:** The following has been included in the methods section: ‘from inception to 16.01.19.’

**R1 Comment 4**

 4. The current information given, it is not sufficiently detailed to be repeatable, for example, the authors could provide more detail of their search strategy, including limits used. Under “Physical focused definitions employed in the review” the authors provide definitions for functional limitation and physical activity. However, they also provide examples of functional limitations, but not physical activity, as physical activity is so broad, to add examples of search words used would add clarity.

**R1 Response to comment 4: **Precise details of both searches are included in Supplementary File 2. In relation to this point, the following has been added in the methods section:

 ‘Physical activity was considered any type of physical training or movement including any form of exercise, physical fitness or therapeutic movement. The full search strategy is available in Supplementary File 2.’

**R1 Comment 5**

 5.The PRISMA diagram is helpful, though it would be more accurate to describe Figure 1 as “PRISMA flow diagram of selection for review”, as it covers the selection/ inclusion process, not the whole review process.

**R1 Response to comment 5: **This has now been labelled as suggested.

**R1 Comment 6**

 6. It might be more appropriate to make reference to Fig 1 earlier in “selection of studies” section.

**R1 Response to comment 6: **Following the scoping review methodology from the Joanna Briggs Institute, Fig. 1 was referenced at the start of the results section. 

**R1 Comment 7**

 7. There was limited description of “data extraction” (it would be useful to hear more about what the specifically designed data extraction sheet contained).

**R1 Response to comment 7: **The following has been added to the methods section: ‘The data extraction instrument collected the following data relating to included studies (author, year of publication, country of study origin, inclusion criteria, living arrangements, physical focused outcomes measured, participant characteristics (number of participants, age, gender, race/ethnicity, percentage with less than high school education, co-morbid conditions), physical focused variables (physical variable measured, type of measure, total number of studies, authors, results), physical activity/sedentary behaviour focused measures (author, type of measure, measure subscale, main results). ‘

**R1 Comment 8**

 Again, limited information provided about data analysis - more detail on what was involved in the narrative synthesis would give greater transparency for the reader.

**Response to R1 Comment 8:** As per point from R2 Comment 19, synthesis of the studies' results was organized under two main headings: Physical functioning and physical activity. As per R1 comment 7 above, details of the data extraction too have also been provided.

**Results**

**R1 Comment 9**

 9. It is shown (in Fig 1) and described (in text) how many studies were excluded at each stage, but apart from the removal of duplicates, reasons for exclusions are not described.

**R1 Response to comment 9:** The reason for exclusion was not containing data relevant to physical functioning limitations or physical activity specific to adults who were homeless. The following has been added to the results section ’After excluding studies which did not containing data relevant to physical functioning limitations or physical activity specific to adults who were homeless, a total of 15 studies were deemed eligible for inclusion in this review.’

**R1** **Comment 10**

 10. Also, the number of studies identified after duplicates removed does not match what is stated in text/Figure 1. (n=2832 in text/ n=2833 in Fig 1).

**R1 Response to comment 10:** The correct value of 2833 is now included in the text.

**R1 Comment 11**

 11. For clarity, it might help to add title and abstracts of to the box “Records screened (n=1815)” in Figure 1.

**Response to comment R1 11:** This has now been inserted into Figure 1.

**R1 Comment 12**

 12. Authors have clearly expressed study characteristics in Table 1. However, it is stated in text they identified 11 quantitative studies and 4 qualitative studies, whereas only 3 studies are explicitly described as qualitative in Table 1. Was it the pilot study by Kendzor et al. (2015) which was also qualitative? If so, it would be useful if the authors made this clear.

**R1** **Response to comment 12:** Apologies for the lack of clarity around this. Two studies were qualitative (Bazari and Quine) and Table 1 has divided studies into quantitative and qualitative.

**R1** **Comment 13**

 13. In Table 1, it would also be useful to be clearer about which studies addressed physical function, which addressed physical activity and which addressed both. For example, where Table 4 describes studies using physical activity measures, Gregg and Bedard (2016) and Marmolejo et al. (2018) feature, but in Table 1 the physical focused outcomes column does not clearly describe physical activity measures (for Gregg and Bedard (2016) exercise and intention to exercise are stated, but is not a measure of actual physical activity levels and Marmolejo et al. (2018) only a flexibility measure is described).

**Response to R1 comment 13:** To improve clarity, a column ‘Physical-functioning measure or Physical activity ****measure’ construct measured has now been added to Table 1. Table 3 now includes Physical Functioning measures included in the review and Table 4 includes Physical Activity measures included in the review.

**R1 Comment 14**

 14 **. **Only minor, but in Table 2, there is slight inconsistency in presentation of data in “gender” column. Sometimes % and numbers, but sometimes only %, is this because numbers were not always provided in studies? If so, you could use “NS”, like you have in other parts of that table.

**Response to R1 comment 14:** NS has been added as suggested.

**R1 Comment 15**

 15. Also, there are typos on p 8, para 1, lines 2-3: “many people homeless experiencing homelessness” and p 10, para 2, line 4: in this study is was”- replace “is” with “it”. Due to the authors not formally assessing methodological quality of the studies, they were limited in how well they could acknowledge biases across studies.

**Response to R1 comment 15**: Typos have been amended. This is correct regarding inability to acknowledge biases across studies. Biases were not formally assessed based on Joanna Briggs methodology for scoping studies which was followed in this review.

 Discussion and Conclusion

**R1 Comment 16**

 16. A clear and succinct summary of the main findings and their implications for understanding the knowledge base, clinical practice and future research. Strengths and limitations of the paper appropriately identified and described.

**Response to comment R1 16:** Thank you to Reviewer 1 for these comments.
